# Ultrafast preparation and detection of entangled atoms

**DOI:** 10.1126/sciadv.abq8227

**Published:** 2023-09-08

**Authors:** Sebastian Eckart, Daniel Trabert, Jonas Rist, Angelina Geyer, Lothar Ph. H. Schmidt, Kilian Fehre, Maksim Kunitski

**Affiliations:** Institut für Kernphysik, Goethe-Universität Frankfurt am Main, Max-von-Laue-Straße 1, 60438 Frankfurt am Main, Germany.

## Abstract

Atoms can form a molecule by sharing their electrons in binding orbitals. These electrons are entangled. Is there a way to break a molecular bond and obtain atoms in their ground state that are spatially separated and still entangled? Here, we show that it is possible to prepare these spatially separated, entangled atoms on femtosecond time scales from single oxygen molecules. The two neutral atoms are entangled in the magnetic quantum number of their valence electrons. In a time-delayed probe step, we use nonadiabatic tunneling, which is a magnetic quantum number–sensitive ionization mechanism. We find a fingerprint of entanglement in the measured ionization probability as a function of the angle between the light’s quantization axis and the molecular axis. This establishes a platform for further experiments that harness the time resolution of strong-field experiments to investigate spatially separated, entangled atoms on femtosecond time scales.

## Teaser

Highly intense laser pulses can prepare and probe spatially separated, entangled atoms on femtosecond time scales.

## INTRODUCTION

Tunneling (*1*–*4*) and entanglement (*5*–*8*) are two of the most intriguing phenomena of quantum mechanics. Entanglement has far-reaching consequences, which were questioned by Einstein, calling it “spooky action at a distance” (*9*, *10*). The realization that the quantum realm violates local realism was groundbreaking (*11*–*13*) and gave rise to technologies that harness entanglement for quantum information protocols (*14*).

In the present work, we want to observe naturally occurring entanglement as it is present in everyday life. As atoms form molecules, electrons become entangled in molecular orbitals. It is not obvious whether the electrons can remain entangled if a molecule dissociates. To address this question, we chose oxygen molecules as an everyday life quantum mechanical system for our studies. We dissociate single oxygen molecules and probe the entanglement of electrons in the two atoms at the time when they are spaced 15 nm apart.

As a tool to probe the properties of the entangled pair of spatially separated atoms, we use highly intense femtosecond laser pulses. The strong laser field can liberate an electron from single atoms by tunnel ionization as the laser field modifies the binding potential generating a transient tunnel barrier. Because the laser pulse is applied to an entangled pair of spatially separated atoms, an ionization of one of the two atoms is expected to affect the wave function of the other atom as well.

Famous examples of entangled states are so-called Bell states, probably most well known in the form of entangled photon pairs (*15*, *16*). In spatially separated atoms, Bell states are routinely prepared by resonant optical transitions (*17*). In this case, the spectral width of the resonant transition limits the speed of preparation. In contrast, we show that Bell-like states can be prepared from single oxygen molecules in their ground state on femtosecond time scales. Further, we present a scheme to probe the quantum properties of the entangled atoms, which is based on nonadiabatic tunnel ionization (*18*). Because tunneling is an inherently fast process that occurs on attosecond time scales (*4*, *19*), this allows us to extend the study of spatially separated, entangled atoms to the ultrafast regime.

**Fig. 1.**
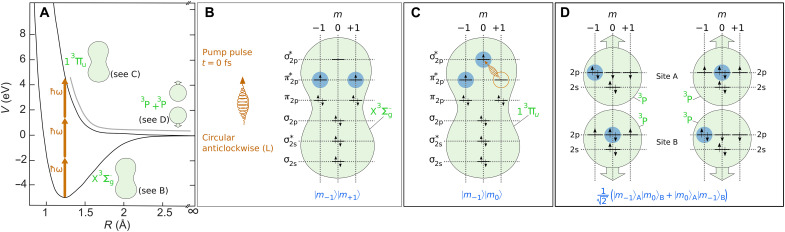
Ultrafast preparation of an atomic Bell-like state. (**A**) Oxygen molecule in its ground state X^3^Σ*_g_* is excited by three-photon absorption from a circularly polarized laser pulse (photons are labeled with ℏω) at a central wavelength of 390 nm and an intensity of 1.0 × 10^14^ W/cm^2^ at the time *t* = 0 fs. This triggers dissociation of the oxygen molecule via the 1^3^Π*_u_* state and results in two neutral oxygen atoms. Panels (B to D) show the same as (A) but in more detail. (**B**) Oxygen molecules in their ground state X^3^Σ*_g_* contain one electron with *m* = −1 and one electron with *m* = +1 in the $\pi_{2p}^{*}$ orbital. (**C**) Circularly polarized pump pulse with an anticlockwise-rotating electric field [indicated by “(L)” in (B)] vector excites the electron from $\pi_{2p}^{*}$ (with *m* = +1) to $\sigma_{2p}^{*}$ (with *m* = 0) by three-photon absorption. This leads to the 1^3^Π*_u_* state that contains an excess electron with a magnetic quantum number of *m* = −1. (**D**) Upon dissociation, this produces two oxygen atoms in their ground state, which have defined magnetic quantum numbers [see blue filled circles in (B) to (D) and note that the indicated electron spins illustrate exemplarily cases only] (see Materials and Methods and Supplementary Materials for details). There is no way to tell which atom has which magnetic quantum number. This results in an entangled, Bell-like state, which is formed by one oxygen atom in “site A” and one oxygen atom in “site B.” A pump pulse with anticlockwise-rotating electric field predominantly prepares an entangled state, which can be written as $|{\text{Ψ}}_{-00-}^{+}\rangle =\frac{1}{\sqrt{2}}({|m_{-1}\rangle }_{A}{|m_{0}\rangle }_{B}+{|m_{0}\rangle }_{A}{|m_{-1}\rangle }_{B})$.

We examine isolated oxygen molecules in the gas phase and perform our studies using cold target recoil ion momentum spectroscopy (COLTRIMS) (*20*), a measurement technique that provides access to the correlated momenta of reaction products from single molecules. The experimental scheme for the ultrafast preparation of a Bell-like state, which is formed by spatially separated atoms, is illustrated in Fig. 1. We prepare spatially separated, entangled atoms by splitting single oxygen molecules in their ground state using a femtosecond laser pulse (pump pulse). It should be noted that the electrons in ground state oxygen molecules are naturally entangled. However, the spatial dimensions of the entangled system are defined by the size of the molecule (~0.1 nm). In our experiment, the pump pulse breaks the molecular bond by three-photon excitation, which triggers the dissociation of the oxygen molecule into two neutral oxygen atoms in their ground state (see Fig. 1). The two atoms move in opposite directions at a velocity of about 5000 m/s. Despite their spatial separation, the two atoms are still entangled in the magnetic quantum number *m* of their valence electrons. We use the following notation for the Bell-like state that is predominantly prepared for a pump pulse with an anticlockwise rotating electric field

$$|{\text{Ψ}}_{-00-}^{+}\rangle =\frac{1}{\sqrt{2}}({|m_{-1}\rangle }_{A}{|m_{0}\rangle }_{B}+{|m_{0}\rangle }_{A}{|m_{-1}\rangle }_{B})$$ (1)

**Fig. 2.**
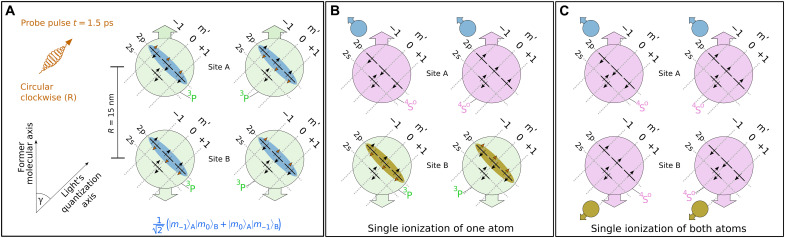
Projection of the prepared Bell-like state to a new basis and tunnel ionization. (**A**) Case in which a molecule dissociates at an angle of γ with respect to the light’s propagation axis is illustrated. At *t* = 1.5 ps, a circularly polarized probe pulse at an intensity of 4.5 × 10^14^ W/cm^2^ hits the dissociating molecule. The laser pulse has a clockwise-rotating electric field [indicated by “(R)”] and projects the magnetic quantum number *m* in the molecular frame onto a new basis *m*′, as illustrated by the arrows within the blue shaded area. The fourth electron that is indicated by orange arrows is in a superposition of all three *m*′ states and entangled with the corresponding electron at the other site. The entanglement of these two electrons in the basis m is indicated by $\frac{1}{\sqrt{2}}({|m_{-1}\rangle }_{A}{|m_{0}\rangle }_{B}+{|m_{0}\rangle }_{A}{|m_{-1}\rangle }_{B})$. (**B**) At site A, nonadiabatic tunnel ionization occurs, which strongly prefers electrons with *m*′ = −1 and acts like a polarizer that projects the wave function on eigenstates that are defined by the new quantization axis. Single ionization at site A instantaneously affects the wave function at site B (ocher colored area). (**C**) With a certain probability, a second electron with *m*′ = −1 is liberated at site B by a sequential tunneling process, such that both oxygen atoms are singly ionized.

Here, ∣*m*_−1_⟩_A_ indicates that, in the atom at site A, there are two electrons with a magnetic quantum number of *m* = −1 in the 2p orbital, while there is only one electron with *m* = 0 and one electron with *m* = +1 (see Fig. 1). The other notations are analogous. The quantization axis of the angular momentum after preparation is defined by the former molecular axis, which is experimentally accessible from the velocity vectors of the detected oxygen atoms (*21*). For an anticlockwise rotating electric field, the $|{\text{Ψ}}_{-00-}^{+}\rangle$ state is not prepared exclusively, but a certain part of the probability density is also prepared in the Bell-like state $|{\text{Ψ}}_{+00+}^{+}\rangle =\frac{1}{\sqrt{2}}({|m_{+1}\rangle }_{A}{|m_{0}\rangle }_{B}+{|m_{0}\rangle }_{A}{|m_{+1}\rangle }_{B})$. Further, it should be noted that the probabilities to prepare $|{\text{Ψ}}_{-00-}^{+}\rangle$ and $|{\text{Ψ}}_{+00+}^{+}\rangle$ depend on the angle between the molecular axis and the light propagation axis. The angle between these two quantization axes is indicated by γ (see discussion of Fig. 2). Because of symmetry, both Bell-like states occur with equal probabilities if the two quantization axes are perpendicular to each other (see the Supplementary Materials for details).

Using a second, time-delayed laser pulse (probe pulse), we investigate the properties of the neutral oxygen atoms and thus obtain information on the entanglement of their electrons. Here, we make use of the fact that strong laser fields can liberate an electron from an atom by tunnel ionization. The laser field modifies the binding potential, generating a transient tunnel barrier that varies on time scales of the laser light’s frequency. For circularly polarized light, this tunnel barrier rotates in the polarization plane, which leads to nonadiabatic dynamics in the classically forbidden region (*18*). These dynamics result in a substantial dependence of the tunneling probability on the electron’s magnetic quantum number *m* (*22*, *23*). Consequently, nonadiabatic tunneling acts as a polarization filter preferring electrons with *m* = −1 for transmission through the tunnel (*24*).

## RESULTS

### Preparation of spatially separated entangled atoms on femtosecond time scales

If one applies a circularly polarized pump pulse with anticlockwise rotating electric field in our experiment, then this excites the $\pi_{2p}^{*}$ electron with *m* = +1 to the $\sigma_{2p}^{*}$ level with *m* = 0. Subsequently, the molecular 1^3^Π*_u_* state dissociates into two oxygen atoms in their ground state (*25*, *26*). The kinetic energy release is centered at 4 eV for this dissociation channel. After the atoms are spatially separated, they are in ^3^P states as exemplarily illustrated in Fig. 1D (see Materials and Methods for details regarding the preparation by the pump pulse and fig. S1 for experimental data, which proves that neutral oxygen atoms are produced by the pump pulse) (*27*). Because of the conservation of angular momentum and the helicity of the pump pulse, the sign of *m* is defined. Our convention regarding the sign of *m* is chosen, such that electrons with *m* = −1 would be counter rotating in a semiclassical picture with respect to the rotational direction of the electric field of the probe pulse (*22*, *24*, *28*). Note that not only we will conduct the experiment with pump pulses that have an anticlockwise rotating electric field, but we will also use clockwise rotating electric fields. In the latter case, the signs of the magnetic quantum numbers of the prepared Bell-like state will be inverted, which leads to the predominant preparation of the state $|{\text{Ψ}}_{+00+}^{+}\rangle =\frac{1}{\sqrt{2}}({|m_{+1}\rangle }_{A}{|m_{0}\rangle }_{B}+{|m_{0}\rangle }_{A}{|m_{+1}\rangle }_{B})$.

The left and right half of Fig. 1D together illustrate the entangled atomic state from Eq. 1. Only 100 fs after the pump pulse has triggered dissociation, the two neutral atoms are separated by about 1 nm, which is far enough to safely neglect classical interaction between them (*29*). After preparation by the pump pulse, the quantization axis of the separated oxygen atoms is given by the former molecular axis. For the sake of completeness, it should be noted that the electron spins that are indicated in Fig. 1 by small vertical arrows only illustrate exemplarily cases. Please see the Supplementary Materials for details on the description of the dissociative 1^3^Π*_u_* state that includes the spin quantum number. However, because nonadiabatic tunnel ionization is an *m*-selective ionization mechanism, the preparation and detection of entangled atoms can be modeled using only the *m* quantum number.

### Investigations of spatially separated atoms by *m*′-selective tunneling

To probe the entanglement of the two spatially separated oxygen atoms in an experiment, one needs an *m*-selective interaction. Nonadiabatic tunnel ionization not only is an ultrafast process that occurs on attosecond time scales (*4*, *19*) but is also *m* selective. In all cases, we use a circularly polarized light field with clockwise rotating electric field as a probe pulse and exploit that nonadiabatic tunneling strongly prefers (*22*, *24*) electrons with *m* = −1. The probe pulse is applied 1.5 ps after the pump pulse. At this time, the two entangled oxygen atoms have a distance of about 15 nm (see Fig. 2A). When the first electron is liberated by the probe pulse, the prepared ionic state is projected to the quantization axis of the probe pulse, which points along the light propagation direction. Strikingly, this projection not only occurs for the atom from which the first electron is liberated. The projection to the new basis occurs for the other spatially separated atom as well. Consequently, the probability to ionize both atoms can carry a fingerprint of entanglement. To test the quantum properties of the state that is prepared by the pump pulse, we study different orientations of the molecular axis with respect to the light propagation axis. The angle between these two quantization axes is indicated by γ (see Fig. 2A). The coincident detection of all charged fragments with our COLTRIMS reaction microscope (*20*) allows us to measure γ and the kinetic energy release (KER) for each dissociation event. This allows us to post-select events that dissociated via the 1^3^Π*_u_* state by restricting the KER to about 4 eV.

As stated above, the quantization axis of the prepared Bell-like state is given by the former molecular axis. When the probe pulse liberates the first electron, the quantization axis for the tunneling process is defined by the light propagation axis (see Fig. 2, A and B). Accordingly, the probe pulse leads to the projection of the prepared Bell-like state to a new set of magnetic quantum numbers, which are labeled with *m*′. Thus, the tunneling process that is driven by the probe pulse is ∣*m*′_−1_⟩ selective (see fig. S3 for experimental data that prove that the probe step is ∣*m*′_−1_⟩ selective). The projection to the new basis can be expressed using the following definitions

$${|m_{-1}\rangle }_{A}:=a{|m_{-1}^{\prime }\rangle }_{A}+b{|m_{0}^{\prime }\rangle }_{A}+c{|m_{+1}^{\prime }\rangle }_{A}$$ (2)

$${|m_{0}\rangle }_{A}:=d{|m_{-1}^{\prime }\rangle }_{A}+e{|m_{0}^{\prime }\rangle }_{A}+f{|m_{+1}^{\prime }\rangle }_{A}$$ (3)

The definitions for ∣*m*_−1_⟩_B_ and ∣*m*_0_⟩_B_ are analogous. The coefficients *a*, *b*, *c*, *d*, *e*, and *f* are scalar values that depend on the angle γ (see fig. S2). The Bell-like state from Eq. 1 can be expressed using the new basis

$$|{\text{Ψ}}_{-00-}^{+}\rangle =\frac{1}{\sqrt{2}}[(a{|m_{-1}^{\prime }\rangle }_{A}+b{|m_{0}^{\prime }\rangle }_{A}+c{|m_{+1}^{\prime }\rangle }_{A})(d{|m_{-1}^{\prime }\rangle }_{B}+e{|m_{0}^{\prime }\rangle }_{B}+f{|m_{+1}^{\prime }\rangle }_{B})+(d{|m_{-1}^{\prime }\rangle }_{A}+e{|m\prime_{0}\rangle }_{A}+f{|m_{+1}^{\prime }\rangle }_{A})(a{|m_{-1}^{\prime }\rangle }_{B}+b{|m_{0}^{\prime }\rangle }_{B}+c{|m_{+1}^{\prime }\rangle }_{B})]$$ (4)

### Observables that shows a fingerprint of entanglement in strong-field ionization

In the following, we distinguish two cases. In the first case, only one of the two atoms is ionized by the probe pulse as illustrated in Fig. 2B. In the second case, the second atom is ionized as well (see Fig. 2C). In both cases, we infer the alignment of the former molecular axis from the momentum vector of the first detected ion (see Materials and Methods for details). This allows us to determine the angle γ between the two quantization axes. After the pump pulse has triggered dissociation, the former molecular axis becomes the quantization axis of the dissociating molecule. When the probe pulse arrives, the light’s propagation direction suddenly becomes the quantization axis for the tunneling process. (Please note that pump and probe pulse have the same propagation direction.)

**Fig. 3.**
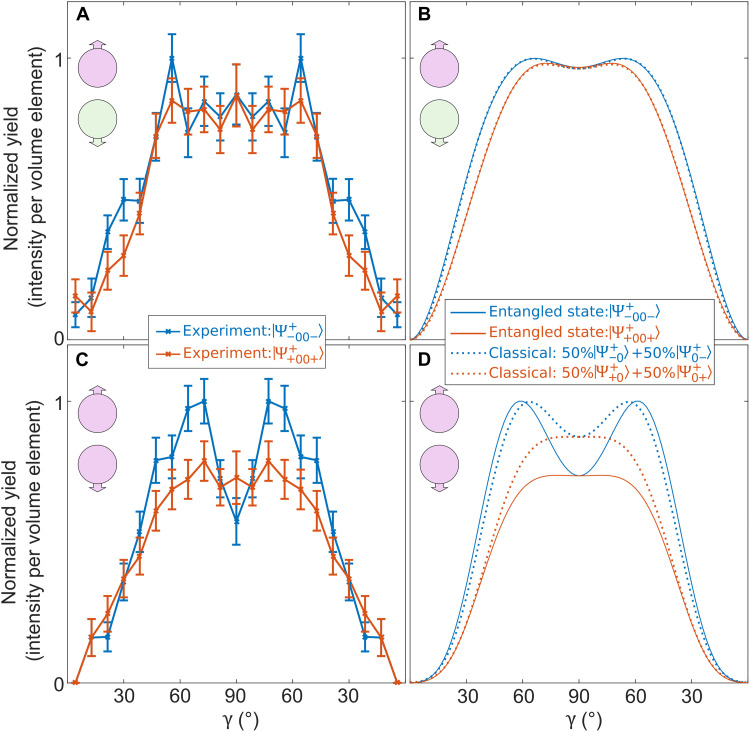
Strong-field ionization of two different Bell-like states. (**A**) Experimental result for the ionization of one of the two atoms. The blue curve shows the ionization probability as a function of γ after predominantly preparing the entangled state $|{\text{Ψ}}_{-00-}^{+}\rangle =\frac{1}{\sqrt{2}}({|m_{-1}\rangle }_{A}{|m_{0}\rangle }_{B}+{|m_{0}\rangle }_{A}{|m_{-1}\rangle }_{B})$. The red curve shows the same for the entangled state $|{\text{Ψ}}_{+00+}^{+}\rangle =\frac{1}{\sqrt{2}}({|m_{+1}\rangle }_{A}{|m_{0}\rangle }_{B}+{|m_{0}\rangle }_{A}{|m_{+1}\rangle }_{B})$. (**B**) Fit of our model that uses the entangled states $|{\text{Ψ}}_{-00-}^{+}\rangle$ and $|{\text{Ψ}}_{+00+}^{+}\rangle$ to the data from (A) is indicated with solid lines. The dotted lines show the fit using a classical state, which is described by $|{\text{Ψ}}_{-0}^{+}\rangle ={|m_{-1}\rangle }_{A}{|m_{0}\rangle }_{B}$ in 50% of the cases and otherwise by $|{\text{Ψ}}_{0-}^{+}\rangle ={|m_{0}\rangle }_{A}{|m_{-1}\rangle }_{B}$ (the notations for $|{\text{Ψ}}_{+0}^{+}\rangle$ and $|{\text{Ψ}}_{0+}^{+}\rangle$ are analogous). (**C** and **D**) Same as (A) and (B) but for the single ionization of both of the two atoms. The results in (D) are obtained by using the models’ parameters that were found for (B) (see "Observables that shows a fingerprint of entanglement in strong field ionization"). The model that uses entangled states shows superior agreement with the experiment compared to the model that uses classical states. Intensity per volume element indicates that the measured yield has been divided by sin(γ). The experimental data have been symmetrized. Error bars show the SD of the statistical error only.

Figure 3A shows the experimental result for the single ionization of one of the two atoms as a function of γ. The blue line shows the result after predominantly preparing $|{\text{Ψ}}_{-00-}^{+}\rangle$, which is the state that is illustrated in Fig. 1D. We have also measured the result for the corresponding state that is produced by flipping the helicity of the pump pulse. This predominantly prepares the energetically degenerate entangled state $|{\text{Ψ}}_{+00+}^{+}\rangle =\frac{1}{\sqrt{2}}({|m_{+1}\rangle }_{A}{|m_{0}\rangle }_{B}+{|m_{0}\rangle }_{A}{|m_{+1}\rangle }_{B})$. It should be noted that the ratio of the probabilities to prepare $|{\text{Ψ}}_{-00-}^{+}\rangle$ or $|{\text{Ψ}}_{+00+}^{+}\rangle$ is a function of γ and described by ${\tilde{q}}_{\mathrm{eff}}(\gamma )$ (see eqs. S8 and S9 in the Supplementary Materials). For γ = 90°, the molecular axis is perpendicular to the light’s propagation direction; thus, $|{\text{Ψ}}_{-00-}^{+}\rangle$ and $|{\text{Ψ}}_{+00+}^{+}\rangle$ are prepared with equal probabilities (see discussion of ${\tilde{q}}_{\mathrm{eff}}$ in the Supplementary Materials for details on how the contributions of $|{\text{Ψ}}_{-00-}^{+}\rangle$ and $|{\text{Ψ}}_{+00+}^{+}\rangle$ are modeled quantitatively). The experimental result is shown as red line in Fig. 3A.

The experimental results for the ionization of one of the two atoms are reproduced by our model that uses entangled states (solid lines in Fig. 3B) and classically correlated states (dashed lines in Fig. 3B). Both models take *m*′-selective tunneling, the absolute ionization probability, the population of the two different states as a function of the molecular orientation, and the angle-dependent dissociation probability into account. Both models have four free parameters that are optimized independently for each model, such that the results in Fig. 3B match the experimental results in Fig. 3A (see the Supplementary Materials for details).

Figure 3C shows the experimental result for the ionization of both oxygen atoms in full analogy to Fig. 3A. The result for $|{\text{Ψ}}_{-00-}^{+}\rangle$ shows a clear double-hump structure. In contrast, the result for $|{\text{Ψ}}_{+00+}^{+}\rangle$ shows no significant double-hump structure, given the statistical error. Figure 3D shows the theoretical results for the single ionization of both atoms using the same two models as for Fig. 3B. Note that all free parameters of the two models are determined using solely the experimental results for the single ionization of one of the atoms (see the Supplementary Materials for details). In other words, the results for the single ionization of one of the atoms is used to characterize the preparation step by the pump pulse (parameters η, β, and κ) and the absolute ionization probability during the probe step (parameter *p*_−_). Thus, the comparison of (C) and (D) of Fig. 3 can be used to benchmark the predictive power of the two models. It is evident that the model that takes entanglement and interference into account (solid lines in Fig. 3D) agrees with the experimental result (Fig. 3C). For comparison, the dashed lines in Fig. 3D show the result from our classical model that includes classical correlations only. It can be seen that the classical model exhibits a less pronounced double-hump structure and a less pronounced difference of the total yield.

## DISCUSSION

Figure 3 shows that there exist fingerprints of entanglement for the strong-field ionization of spatially separated atoms. It is found that the two models lead to different predictions (see Fig. 3D) regarding an experimentally accessible quantity (see Fig. 3, C and D). This shows that there are observables in strong-field ionization that allow one to distinguish entangled states from classically correlated states.

### A quantum-mechanical thought experiment reveals the origin of the observed fingerprints

In the following, we will present an explanation why there are observables that carry a fingerprint of entanglement in our experiment. To this end, we perform a quantum-mechanical thought experiment and apply the probe pulse only at site A (in contrast to bound molecules, this is possible in our case because the two atoms are spatially separated). Let us further assume that we could ionize all parts of the wave function at site A that are in the ∣*m*′_−1_⟩_A_ state and could not ionize any other part of the wave function. Then, the single ionization probability would be given by $\frac{1}{2}(a^{2}+d^{2})$ (see fig. S2 for details). Because of entanglement, ionization at site A would result in a modified wave function for the singly ionized Bell-like state as illustrated in Fig. 2B. In a next step, we could use a second probe pulse that is applied at site B, which can lead to the subsequent ionization of the remaining atom. This would, with a certain probability, produce two singly charged oxygen ions (see Fig. 2C). The quantum-mechanical prediction for the probability to liberate one electron with $|m_{-1}^{\prime }\rangle$ at site A and one electron with $|m_{-1}^{\prime }\rangle$ at site B is given by $\frac{1}{2}{\left(ad+da\right)}^{2}$ = 2*a*^2^*d*^2^ (see table S1 for details). This finding, which is based on Eq. 4, is in contrast to the expectation of any classical theory based on local realism.

For comparison, we can redo this though experiment using a classically correlated state, which is described by $|{\text{Ψ}}_{-0}^{+}\rangle ={|m_{-1}\rangle }_{A}{|m_{0}\rangle }_{B}$ in 50% of the cases and by $|{\text{Ψ}}_{0-}^{+}\rangle ={|m_{0}\rangle }_{A}{|m_{-1}\rangle }_{B}$ in the other 50% of the cases. Again, we assume that we liberate all electrons that are in the ∣*m*′_−1_⟩ state at site A. This results in an ionization probability for the atom at site A that is given by $\frac{1}{2}a^{2}+\frac{1}{2}d^{2}$, which is identical to the quantum-mechanical expectation. Strikingly, the probability to ionize both atoms is $\frac{1}{2}{\left(ad\right)}^{2}+\frac{1}{2}{\left(da\right)}^{2}$ = *a*^2^*d*^2^ for the classically correlated state, which differs compared to the prediction for the entangled state. This is a key finding and illustrates why the probability to ionize both atoms is different for entangled and classically correlated atoms. This provides a microscopic explanation for the observed fingerprints of entanglement in strong-field ionization. In short, it is the description of the prepared state as an entangled state that captures the correlations of the wave function’s amplitudes. Thus, ionization of the first atom leads to a projection of the entangled state to new eigenstates, which eventually alters the wave function of the other atom (see Fig. 2, B and C). Consequently, the experimentally accessible probability to ionize both atoms is modified in a way that cannot be explained by classical correlations (see Fig. 3, C and D).

The entanglement of the two atoms is very stable (see the Supplementary Materials for the internal dynamics in the two atoms that are due to spin-orbit splitting). The reason for this is that the two neutral atoms are in their ground state. The phase of the Bell-like state $\frac{1}{\sqrt{2}}({|m_{-1}\rangle }_{A}{|m_{0}\rangle }_{B}+e^{i\phi }{|m_{0}\rangle }_{A}{|m_{-1}\rangle }_{B})$ can only be changed with an *m*-selective interaction that is different at site A and site B (see Materials and Methods for further details on the phase of the Bell-like state). For example, an (spatially inhomogeneous) electric field would not change the relative phase ϕ. The only external field that might affect ϕ is a spatially inhomogeneous magnetic field. This would shift the energy levels as a function of the magnetic quantum number in site A and site B differently. So, if one applied a magnetic field of 1 mT at site A and 0 mT at site B, then this would lead to an energy difference of the ∣*m*_−1_⟩_A_ and the ∣*m*_0_⟩_A_ state of about Δ*E* = μ*_B_* · 1 mT =58 neV. The time constant that belongs to this energy splitting is 71 ns. In addition, this is a very conservative estimate of the inhomogeneity of our magnetic field because the overall magnetic field in our spectrometer is 1.01 mT. We expect that inhomogeneities of the magnetic field are on the order of 5% within a volume of several cubic centimeters. Thus, the inhomogeneity of the magnetic field on the length scale that is defined by the distance of the two atoms (15 nm) will be on the order of 0.05 mT or even (much) better. Accordingly, we expect that phase ϕ is stable for several microseconds or even longer.

We have conducted an experiment with spatially separated atoms on a femtosecond time scale for which entanglement is relevant. The entangled states are prepared using a circularly polarized pump pulse that triggers the dissociation of molecular oxygen. Subsequently, the quantization axis of the prepared state is given by the former molecular axis. A time-delayed, circularly polarized probe pulse ionizes one or both of the two atoms. The angle between the molecular axis and the light propagation axis of the probe pulse, γ, governs the projection of the prepared state to a new basis. This projection affects the wave function of both atoms. We find that it is possible to model the probability to ionize one of the two entangled atoms without taking entanglement into account. The expected probability to ionize both atoms as a function of γ differs for an entangled state and a classically correlated state. This theoretical result illustrates that there are observables in strong-field ionization experiments that can be used to distinguish if atoms are entangled or only classically correlated. This might inspire further theoretical approaches using more sophisticated models or even ab initio simulations. Our experiment shows better agreement with the model that includes entanglement. This demonstrates that there are observables in strong-field tunnel ionization that are sensitive to the relative phase between the wave functions of spatially separated atoms. In addition, our experiment shows that the entanglement, which is due to a covalent bond in a molecule, can survive the dissociation in a strong laser field. The ultrafast nature of tunnel ionization itself, which occurs on attosecond time scales, paves the way toward time-resolved studies of entangled states. Last, our findings highlight the importance of entanglement in chemical systems (*30*, *31*) and in multielectron processes on attosecond time scales (*8*, *32*) and contribute to recent investigations of the nonclassical nature of strong-field processes (*33*–*35*).

## MATERIALS AND METHODS

### Laser setup

The optical setup is based on a laser that operates at a central wavelength of 780 nm [KMLabs Dragon, 40-fs full width at half maximum (FWHM), 8 kHz]. The laser pulses that are used as pump pulses are frequency doubled in a 200-μm beta barium borate crystal producing laser pulses at a central wavelength of 390 nm. The probe pulses have a central wavelength of 780 nm. The intensity, the ellipticity, and the main axis of the polarization ellipse of both pulses can be adjusted independently for pump and probe pulses. Both pulses were circularly polarized. The pump-probe delay of 1.5 ps was set using a micrometer delay stage. Both laser pulses were focused by a spherical mirror (*f* = 80 mm) onto a cold supersonic jet of molecular oxygen, which was created by expanding oxygen gas through a 30-μm-diameter nozzle into vacuum. The helicity of the circularly polarized pump pulse was inverted every 2 min to minimize systematic errors. In Fig. 1, the case of a pump pulse with anticlockwise-rotating electric field is illustrated. Throughout the experiment, the helicity of the probe pulses was not changed and a circularly polarized pulse with clockwise-rotating electric field was used. The optical setup is the same as in (*24*). The intensity of the pump pulse of 1.0 × 10^14^ W/cm^2^ was calibrated from the shift of the above threshold ionization peaks as a function of the laser intensity, which is due to the change in ponderomotive energy. The intensity of the probe pulses at a central wavelength of 780 nm was obtained from the measured drift momentum of the electron and found to be 4.5 × 10^14^ W/cm^2^. The uncertainty of the absolute intensity for the pump and the probe pulse is estimated to be 20%.

### Particle detection

We use a COLTRIMS reaction microscope (*20*) to detect up to two singly charged oxygen ions in coincidence with one electron. The electron and ion arm of the spectrometer have a length of 378 and 67.8 mm, respectively. The charged fragments are guided by a homogeneous electric field (17.3 V cm^−1^) and a homogeneous magnetic field (10.1 G) toward time- and position-sensitive detectors. Each detector consists of a stack of two multichannel plates (MCPs). The MCPs of the electron and the ion detector have a diameter of 120 and 80 mm, respectively. For both detectors, the MCP stack is followed by a three-layer hexagonal delay line anode (HEX) (*36*). We found that for *p_y_* < 0 a.u. (atomic units) the electron detection efficiency was affected by a local inefficiency of the MCP. To minimize systematic errors due to detector inefficiencies, we excluded events with an electron momentum *p_y_* < 0 a.u. (the gas jet propagates along the *p_y_* direction). The molecular axis is accessible by making use of the axial recoil approximation, which holds in our case because the dissociation is fast compared to the rotation of the 1^3^Π*_u_* state. Therefore, we infer the former molecular axis from the momentum of the detected ion, which points along the molecular axis at the time the molecule is hit by the pump pulse (*21*).

### Preparation of the Bell-like state by the pump pulse

The circularly polarized pump pulse at a central wavelength of 390 nm excites the oxygen molecule and thereby leads to dissociation via the 1^3^Π*_u_* state as shown in Fig. 1. The expected KER of the two ^3^P oxygen atoms is 4 eV according to energy conservation for a three-photon absorption. The curves shown in Fig. 1A are taken from (*25*).

Inspection of Fig. 2 form (*25*) reveals that excitation to the 1^3^Π*_u_* state is the only few-photon transition that is allowed, taking the Franck-Condon principle, energy conservation, and parity selection rules into account. In particular, two-photon transitions from the ground state to the C^3^Δ*_u_* state or the A^3^${\text{Σ}}_{u}^{+}$ state are forbidden because parity must not change for a two-photon transition. In full analogy, three-photon transitions from the ground state to the 1^3^Π*_g_* state are also forbidden. Further, three-photon transitions from the ground state to the B^3^${\text{Σ}}_{u}^{-}$ state are not forbidden but would lead to the atomic states ^1^D + ^3^P and a kinetic energy release of about 2 eV because the ^1^D state is 2 eV above the ground state. This allows for the conclusion that the two spatially separated atoms with a total KER of about 4 eV, which are investigated in our experiment, are both in a ^3^P state, and dissociation occurs via the 1^3^Π*_u_* state (see Fig. 1).

The ground state of molecular oxygen, the X^3^Σ*_g_* state, has an equal number of electrons with *m* = −1 and = +1. The illustration in Fig. 1 uses the quantum numbers of the individual electrons that form the molecular states X^3^Σ*_g_* and 1^3^Π*_u_* via a linear combination of atomic orbitals (LCAOs). Within LCAO, the transition from X^3^Σ*_g_* to 1^3^Π*_u_* corresponds to a transition of an electron from the $\pi_{2p}^{*}$ state with *m* = +1 to the $\sigma_{2p}^{*}$ state with *m* = 0 (as illustrated in Fig. 1C) or to a transition of an electron from the $\pi_{2p}^{*}$ state with *m* = −1 to the $\sigma_{2p}^{*}$ state with *m* = 0. The helicity of the circularly polarized pump pulses defines which of these two transitions is favored [also see discussion of ${\tilde{q}}_{\mathrm{eff}}(\gamma )$ in the Supplementary Materials].

Figure S3 shows that the electron energy spectra contain information on the helicity of the pump pulse [also see (*24*)]. Further, the results from fig. S3 reveal that the quantization axis of the ^3^P oxygen atoms is defined by the former molecule axis until the probe pulse arrives. Depending on the helicity of the circularly polarized pump pulse, the two ^3^P oxygen atoms in their ground state are predominantly prepared in the state $|{\text{Ψ}}_{-00-}^{+}\rangle =\frac{1}{\sqrt{2}}({|m_{-1}\rangle }_{A}{|m_{0}\rangle }_{B}+{|m_{0}\rangle }_{A}{|m_{-1}\rangle }_{B})$ or in the energetically degenerate state $|{\text{Ψ}}_{+00+}^{+}\rangle =\frac{1}{\sqrt{2}}({|m_{+1}\rangle }_{A}{|m_{0}\rangle }_{B}+{|m_{0}\rangle }_{A}{|m_{+1}\rangle }_{B})$.

As shown in Fig. 1 exemplarily, the two atoms are in ^3^P states (*27*). Because of spin-orbit splitting, the energy of the ^3^P states can differ by up to 28.1 meV. This gives rise to spin-orbit dynamics. We have carried out an analysis as for fig. S3 for several pump-probe delays in steps of 20 fs. For the present work, we chose a pump-probe delay *T* of 1533 fs because it produced the maximum contrast comparing left circularly polarized pump pulses to right circularly polarized pump pulses (see the Supplementary Materials and fig. S5B). For the pump-probe delay of maximum contrast, spin-orbit dynamics can be neglected [see fig. S5B and equation 6 in (*24*) and (*37*)]. Taking spin-orbit dynamics into account shows that, in our experiment, 95% of the entangled atoms are described by the $|{\text{Ψ}}_{-00-}^{+}\rangle$ and the $|{\text{Ψ}}_{+00+}^{+}\rangle$ state. To this end, we assume that all relevant pump-probe delays are modeled by a Gaussian distribution with a width (FWHM) of 20 fs (see discussion of fig. S5B). For the sake of simplicity, we assume that 100% of the entangled atoms are described by the $|{\text{Ψ}}_{-00-}^{+}\rangle$ state. Technically, the pump-probe delay of 1533 fs was set by moving the delay stage 230 μm away from the position where there was temporal overlap of pump and probe pulse. We estimate the experimental uncertainty of the manually operated translation stage to be 5 μm, which corresponds to an uncertainty of the absolute pump-probe delay of 33 fs.

### Expression of the Bell-like state using the quantization axis of the probe pulse

Here, we discuss the Bell-like state $|{\text{Ψ}}_{-00-}^{+}\rangle$ (see Eq. 1). The quantization axis of the Bell-like state is given by the quantization axis of the former molecule and the Bell-like state is expressed using the basis ∣*m*_−1_⟩, ∣*m*_0_⟩, and ∣*m*_+1_⟩. If this state is ionized by a circularly polarized probe pulse at a central wavelength of 780 nm, the light propagation axis of the probe pulse defines the new quantization axis, which is described using the basis ∣*m*′_−1_⟩, ∣*m*′_0_⟩, and ∣*m*′_+1_⟩. γ denotes the angle between the two quantization axes (see Fig. 2A). As shown in Eq. 4, the coefficients *a*, *b*, *c*, *d*, *e*, and *f* can be used to express the prepared Bell-like state in the basis of ∣*m*′_−1_⟩, ∣*m*′_0_⟩, and ∣*m*′_+1_⟩. The γ-dependent, real-valued coefficients *a*, *b*, *c*, *d*, *e*, and *f* are defined by Eqs. 2 and 3 (also see fig. S2). Here, ∣*m*_−1_⟩, ∣*m*_0_⟩, and ∣*m*_+1_⟩ are the atomic 2p orbitals with *m* = −1, *m* = 0, and *m* = +1, respectively. Rotation of the states ∣*m*_−1_⟩, ∣*m*_0_⟩, and ∣*m*_+1_⟩ by the angle γ leads to the new basis ∣*m*′_−1_⟩, ∣*m*′_0_⟩, and ∣*m*′_+1_⟩. These conclusions can be drawn for the state $|{\text{Ψ}}_{+00+}^{+}\rangle$ in full analogy.

The singly ionized oxygen atoms are all in the ^4^S^o^ state. The energetically closest, alternatively available final state is the ^2^D^o^ state, which is very unlikely to be populated by strong-field ionization (*3*) because it has an ionization potential that is 3.3 eV higher compared to the ^4^*S*^o^ state. Thus, the ^4^S^o^ state is the only relevant final state for the singly charged oxygen ions. All 2p electrons in the ^4^S^o^ state must have the same spin, which justifies the notation in Figs. 1 and 2.

### The phase ϕ of the Bell-like state

In principle, there can be any relative phase ϕ between the two parts of the electronic wave function of the Bell-like states $|{\text{Ψ}}_{-00-}^{+}\rangle$ and $|{\text{Ψ}}_{+00+}^{+}\rangle$. For $|{\text{Ψ}}_{-00-}^{+}\rangle$, this results in the expression $\frac{1}{\sqrt{2}}({|m_{-1}\rangle }_{A}{|m_{0}\rangle }_{B}+e^{i\phi }{|m_{0}\rangle }_{A}{|m_{-1}\rangle }_{B})$. In our experiment, the phase ϕ must be close to zero, which leads to *e*^*i*ϕ^ = 1. This is evident from comparing the experimental result with the predictions from the model that uses entangled states for different values of ϕ. As a reference, we use the experimental result (see Fig. 3, A and C). For comparison, we show the theoretic results for ϕ = 45°, ϕ = 90°, and ϕ = 110° in fig. S4. To this end, the definition of the *C* coefficients of our quantum-mechanical model is generalized using the expression $C_{xy}^{\mathrm{phase}}:=\frac{1}{2}{|\langle m_{y}^{\prime }{|{}_{B}\langle m_{x}^{\prime }|}_{A}{|m_{-1}\rangle }_{A}{|m_{0}\rangle }_{B}\;+e^{i\phi }\langle m_{y}^{\prime }{|{}_{B}\langle m_{x}^{\prime }|}_{A}{|m_{0}\rangle }_{A}{|m_{-1}\rangle }_{B}|}^{2}$. This is equivalent to multiplying the second summand in each entry in table S1 with *e*^*i*ϕ^. For each value of ϕ, the data for single ionization (Fig. 3A) are used to find the parameters *p*_−_, η, β, and κ (see the caption of fig.S4 for the corresponding values). For ϕ = 90°, the quantum-mechanical model yields the same result as the classical model (compare dashed lines in Fig. 3, B and D with fig. S4, B and E). It is found that for ∣ϕ∣ < 90° (∣ϕ∣ > 90°), the double-hump structure for double ionization is more (less) pronounced compared to the classical model. Comparison of fig. S4 with the experimental result in Fig. 3 shows that ϕ must be close to zero (please also see the section on spin-orbit dynamics of entangled atoms).

## Supplementary Materials

### This PDF file includes:

Supplementary Text Figs. S1 to S5 Table S1 References

## References

[1] S. L. Chin, F. Yergeau, P. Lavigne, Tunnel ionisation of Xe in an ultra-intense CO_2_ laser field (10^14^ W cm^-2^) with multiple charge creation. J. Phys. B. 18, L213–L215 (1985).

[2] P. B. Corkum, Plasma perspective on strong field multiphoton ionization. Phys. Rev. Lett. 71, 1994–1997 (1993). 10054556 10.1103/PhysRevLett.71.1994

[3] L. V. Keldysh, Ionization in the field of a strong electromagnetic wave. Sov. Phys. JETP. 20, 1307–1314 (1965).

[4] P. Eckle, A. N. Pfeiffer, C. Cirelli, A. Staudte, R. Dörner, H. G. Muller, M. Büttiker, U. Keller, Attosecond ionization and tunneling delay time measurements in helium. Science 322, 1525–1529 (2008). 19056981 10.1126/science.1163439

[5] E. Hagley, X. Maître, G. Nogues, C. Wunderlich, M. Brune, J. M. Raimond, S. Haroche, Generation of Einstein-Podolsky-Rosen pairs of atoms. Phys. Rev. Lett. 79, 1–5 (1997).

[6] J. S. Bell, On the Einstein Podolsky Rosen Paradox. Phys. Phys. Fiz. 1, 195–200 (1964).

[7] E. Schrödinger, Die gegenwärtige Situation in der Quantenmechanik. Naturwissenschaften 23, 807–812 (1935).

[8] M. J. J. Vrakking, Control of attosecond entanglement and coherence. Phys. Rev. Lett. 126, 113203 (2021). 33798339 10.1103/PhysRevLett.126.113203

[9] A. Einstein, B. Podolsky, N. Rosen, Can quantum-mechanical description of physical reality be considered complete? Phys. Rev. 47, 777–780 (1935).

[10] M. Born, Metaphysical conclusions, in *Natural Philosophy of Cause and Chanc*e (Clarendon Press, Oxford, 1949), pp. 122.

[11] J. F. Clauser, A. Shimony, Bell’s theorem. Experimental tests and implications. Rep. Prog. Phys. 41, 1881–1927 (1978).

[12] A. Aspect, P. Grangier, G. Roger, Experimental tests of realistic local theories via Bell’s theorem. Phys. Rev. Lett. 47, 460–463 (1981).

[13] A. Aspect, P. Grangier, G. Roger, Experimental realization of Einstein-Podolsky-Rosen-Bohm Gedankenexperiment: A new violation of Bell's inequalities. Phys. Rev. Lett. 49, 91–94 (1982).

[14] J. I. Cirac, P. Zoller, Quantum computations with cold trapped ions. Phys. Rev. Lett. 74, 4091–4094 (1995). 10058410 10.1103/PhysRevLett.74.4091

[15] S. J. Freedman, F. Clauser, Experimental test of local hidden-variable theories. Phys. Rev. Lett. 28, 938–941 (1972).

[16] J. W. Pan, D. Bouwmeester, H. Weinfurter, A. Zeilinger, Experimental entanglement swapping: Entangling photons that never interacted. Phys. Rev. Lett. 80, 3891–3894 (1998).

[17] Q. A. Turchette, C. S. Wood, B. E. King, C. J. Myatt, D. Leibfried, W. M. Itano, C. Monroe, D. J. Wineland, Deterministic entanglement of two trapped ions. Phys. Rev. Lett. 81, 3631–3634 (1998).

[18] M. Ivanov, M. Spanner, O. Smirnova, Anatomy of strong field ionization. J. Mod. Opt. 52, 165–184 (2005).

[19] U. S. Sainadh, H. Xu, X. Wang, A. Atia-Tul-Noor, W. C. Wallace, N. Douguet, A. Bray, I. Ivanov, K. Bartschat, A. Kheifets, R. T. Sang, I. V. Litvinyuk, Attosecond angular streaking and tunnelling time in atomic hydrogen. Nature 568, 75–77 (2019). 30886392 10.1038/s41586-019-1028-3

[20] J. Ullrich, R. Moshammer, A. Dorn, R. Dörner, L. P. H. Schmidt, H. Schmidt-Böcking, Recoil-ion and electron momentum spectroscopy: Reaction-microscopes. Rep. Prog. Phys. 66, 1463–1545 (2003).

[21] R. M. Wood, Q. Zheng, A. K. Edwards, M. A. Mangan, Limitations of the axial recoil approximation in measurements of molecular dissociation. Rev. Sci. Instrum. 68, 1382–1386 (1997).

[22] I. Barth, O. Smirnova, Nonadiabatic tunneling in circularly polarized laser fields: Physical picture and calculations. Phys. Rev. A 84, 063415 (2011).

[23] T. Herath, L. Yan, S. K. Lee, W. Li, Strong-field ionization rate depends on the sign of the magnetic quantum number. Phys. Rev. Lett. 109, 043004 (2012). 23006084 10.1103/PhysRevLett.109.043004

[24] S. Eckart, M. Kunitski, M. Richter, A. Hartung, J. Rist, F. Trinter, K. Fehre, N. Schlott, K. Henrichs, L. P. H. Schmidt, T. Jahnke, M. Schöffler, K. Liu, I. Barth, J. Kaushal, F. Morales, M. Ivanov, O. Smirnova, R. Dörner, Ultrafast preparation and detection of ring currents in single atoms. Nat. Phys. 14, 701–704 (2018).

[25] R. P. Saxon, B. Liu, Ab initio configuration interaction study of the valence states of O_2_. J. Chem. Phys. 67, 5432–5441 (1977).

[26] H. M. Lambert, A. A. Dixit, E. W. Davis, P. L. Houston, Quantum yields for product formation in the 120-133 nm photodissociation of O_2_. J. Chem. Phys. 121, 10437–10446 (2004). 15549924 10.1063/1.1809114

[27] Y. L. Huang, R. J. Gordon, The multiplet state distribution of O(^3^P_J_) produced in the photodissociation of O_2_ at 157 nm. J. Chem. Phys. 94, 2640–2647 (1991).

[28] I. Barth, J. Manz, Electric ring currents in atomic orbitals and magnetic fields induced by short intense circularly polarized π laser pulses. Phys. Rev. A 75, 012510 (2007).

[29] S. A. Trushin, W. E. Schmid, W. Fu, Time-resolved photodissociation of oxygen at 162 nm. J. Phys. B. 44, 165602 (2011).

[30] L. Ding, S. Mardazad, S. Das, S. Szalay, U. Schollwöck, Z. Zimborás, C. Schilling, Concept of orbital entanglement and correlation in quantum chemistry. J. Chem. Theory Comput. 17, 79–95 (2021). 33430597 10.1021/acs.jctc.0c00559

[31] J. Li, S. Kais, Entanglement classifier in chemical reactions. Sci. Adv. 5, eaax5283 (2019). 31414049 10.1126/sciadv.aax5283 PMC6677555

[32] M. Ruberti, Onset of ionic coherence and ultrafast charge dynamics in attosecond molecular ionisation. Phys. Chem. Chem. Phys. 21, 17584–17604 (2019). 31372608 10.1039/c9cp03074c

[33] J. Rivera-Dean, T. Lamprou, E. Pisanty, P. Stammer, A. F. Ordóñez, A. S. Maxwell, M. F. Ciappina, M. Lewenstein, P. Tzallas, Strong laser fields and their power to generate controllable high-photon-number coherent-state superpositions. Phys. Rev. A 105, 033714 (2022).

[34] M. Lewenstein, M. F. Ciappina, E. Pisanty, J. Rivera-Dean, P. Stammer, T. Lamprou, P. Tzallas, Generation of optical Schrödinger cat states in intense laser–matter interactions. Nat. Phys. 17, 1104–1108 (2021).

[35] A. S. Maxwell, L. B. Madsen, M. Lewenstein, Entanglement of orbital angular momentum in non-sequential double ionization. Nat. Commun. 13, 4706 (2022). 35948552 10.1038/s41467-022-32128-z PMC9365801

[36] O. Jagutzki, A. Cerezo, A. Czasch, R. Dörner, M. Hattaß, M. Huang, V. Mergel, U. Spillmann, K. Ullmann-Pfleger, T. Weber, H. Schmidt-Böcking, G. D. W. Smith, Multiple hit readout of a microchannel plate detector with a three-layer delay-line anode. IEEE Trans. Nucl. Sci. 49, 2477–2483 (2002).

[37] A. N. Pfeiffer, S. G. Sayres, S. R. Leone, Calculation of valence electron motion induced by sequential strong-field ionisation. Mol. Phys. 111, 2283–2291 (2013).

[38] A. V. Baklanov, L. M. C. Janssen, D. H. Parker, L. Poisson, B. Soep, J. M. Mestdagh, O. Gobert, Direct mapping of recoil in the ion-pair dissociation of molecular oxygen by a femtosecond depletion method. J. Chem. Phys. 129, 214306 (2008). 19063560 10.1063/1.3026613

[39] K. Fehre, D. Trojanowskaja, J. Gatzke, M. Kunitski, F. Trinter, S. Zeller, L. P. H. Schmidt, J. Stohner, R. Berger, A. Czasch, O. Jagutzki, T. Jahnke, R. Dörner, M. S. Schöffler, Absolute ion detection efficiencies of microchannel plates and funnel microchannel plates for multi-coincidence detection. Rev. Sci. Instrum. 89, 045112 (2018). 29716368 10.1063/1.5022564

[40] I. Barth, O. Smirnova, Nonadiabatic tunneling in circularly polarized laser fields. II. Derivation of formulas. Phys. Rev. A 87, 013433 (2013).

[41] J. J. Lin, D. W. Hwang, Y. T. Lee, X. Yang, Photodissociation of O_2_ at 157 nm: Experimental observation of anisotropy mixing in the O_2_+hν→O(^3^P)+O(^3^P) channel. J. Chem. Phys. 109, 1758–1762 (1998).

[42] N. B. Delone, V. P. Krainov, AC Stark shift of atomic energy levels. Phys. Usp. 42, 669–687 (1999).

[43] A. H. N. C. De Silva, D. Atri-Schuller, S. Dubey, B. P. Acharya, K. L. Romans, K. Foster, O. Russ, K. Compton, C. Rischbieter, N. Douguet, K. Bartschat, D. Fischer, Using circular dichroism to control energy transfer in multiphoton ionization. Phys. Rev. Lett. 126, 023201 (2021). 33512178 10.1103/PhysRevLett.126.023201

[44] X. Wu, Z. Yang, S. Zhang, X. Ma, J. Liu, D. Ye, Buildup time of Autler-Townes splitting in attosecond transient absorption spectroscopy. Phys. Rev. A 103, L061102 (2021).

[45] M. C. G. N. Van Vroonhoven, G. C. Groenenboom, Reassignment of the O_2_ spectrum just below dissociation threshold based on ab initio calculations. J. Chem. Phys. 117, 5240–5251 (2002).

