# Supplementary material for: Ultrafast preparation and detection of entangled atoms
Source: Sci Adv. 2023 Sep 8;9(36):eabq8227. doi: 10.1126/sciadv.abq8227 (PMC10491222; doi:10.1126/sciadv.abq8227)
Supplement: Supplementary file 1 — Supplementary Text Figs. S1 to S5 Table S1 References [file sciadv.abq8227_sm.pdf]

Science Advances
AAAS

Supplementary Materials for

Ultrafast preparation and detection of entangled atoms

Sebastian Eckart *et al.*

Corresponding author: Sebastian Eckart, eckart@atom.uni-frankfurt.de

Sci. Adv. **9**, eabq8227 (2023)
DOI: 10.1126/sciadv.abq8227

This PDF file includes:

Supplementary Text
Figs. S1 to S5
Table S1
References

Supplementary Text

Experimental details regarding the isolation of the neutral dissociation channel

The pump pulses lead to dissociation of oxygen molecules without ionizing them (see Fig. 1). In our experiment only charged particles are detected. The existence of neutral atoms, that are produced by the pump pulse, is proven using the data that is shown in Fig. S1. This approach is similar to the femtosecond depletion method from Ref. (*38*).

For Fig. S1A only the pump pulses were applied and the kinetic energy release (KER) of the detected ions is shown versus the kinetic energy of the coincidently detected electron. The KER is calculated to be two times the kinetic energy of the first detected oxygen ion, which is a very good approximation for the present experiment.

In strong field ionization, the momentum of the liberated electron is often approximated to be determined by the negative vector potential of the incident light field. The absolute value of the vector potential of the pump pulse is 0.32 a.u. (corresponds to an expected electron energy of 1.4 eV). This is in good agreement with the measured electron energies in Fig. S1A.

Next, we inspect Fig. S1B which shows the same as Fig. S1A but here only the probe pulses are used. The probe pulse’s vector potential is 1.37 a.u. (corresponds to an electron energy of 25 eV). This is also in good agreement with the observed electron’s kinetic energy. This allows for the conclusion that the electron energy can be used to distinguish electrons from the pump and the probe pulse in our pump-probe experiment.

Fig. S1C shows the same as Fig. S1A and S1B but here the pump and the probe pulse are both applied. It is important to note that in Fig. S1C there is an additional peak that is not evident for the pump pulse alone or the probe pulse alone. This peak is at a KER of about 4 eV and an electron energy of about 25 eV. The electron energy of 25 eV indicates that the detected electron must have been liberated by the probe pulse. The low count rates for a KER of 4 eV in the case of the probe pulse alone (or the pump pulse alone) show that the peak at a KER of 4 eV in Fig. S1C is due to molecules that were dissociated to two neutral oxygen atoms with a KER of 4 eV by the pump pulse.

For the sake of completeness, we note that for the cases where only the probe pulse is used, we observe a very pronounced peak at a KER of 7.6 eV, which is due to the production of two singly charged oxygen atoms (not shown in Fig. S1). For these events, the two ions are produced at an internuclear distance that is on the order of 1.3 Å, which is the internuclear distance for the molecular ground state (see Fig. 1A). This leads to significant Coulomb repulsion and the high KER of about 7.6 eV.

As explained above, the KER of about 4 eV is caused by the pump pulse. 1.5 picoseconds later, the probe pulse liberates one or two electrons. At this time the internuclear distance is on the order of 15 nm. Thus, the change of the KER that is due to ionization is negligible. This finding is further illustrated by Fig. S1D. The distributions of the KER in Fig. S1A and S1B are shown as blue and red data points, respectively. The yellow data points show the subset of the data from Fig. S1C for which two singly charged oxygen ions were detected. The green data points also show a subset of the data from Fig. S1C, but here only those events are considered in which one singly charged oxygen ion was detected. For the green data points, random coincidences are subtracted. Here, random coincidences are due to events in which two oxygen ions were produced but only one was detected. This subtraction of random coincidences accounts for the finite detection efficiency of our ion detector, which was determined as in Ref. (*39*) and found to be 55%. The experimental data shown in Fig. 3 is obtained in the same way as the data shown in green and yellow in Fig.

S1D with the difference, that for the data shown in Fig. 3 the background from the pump pulse alone and the probe pulse alone was subtracted after normalization to equal acquisition times. Further, for Fig. 3A (3C) the KER was restricted to 3.6 eV – 4.4 eV (3.7 eV – 4.5 eV). Fig. S3 shows further details regarding the measured electron spectra.

Quantitative modeling of the ionization of one of the two atoms using entangled states

Non-adiabatic tunnel-ionization prefers the liberation of electrons with $|m'_{-1}\rangle$. Let p_- be the probability to liberate an electron in the state $|m'_{-1}\rangle$. For our probe pulse and the ionization potential of atomic oxygen $I_p = 13.62$ eV, the tunneling probability (see Eq. 108 and 109 in Ref. (*40*)) for an electron with $|m'_{+1}\rangle$ is $p_+ \approx 0.4 \cdot p_-$ and the tunneling probability for an electron with $|m'_0\rangle$ is $p_0 \approx 0.05 \cdot p_-$.

The Bell-like state is described by a two-electron wave function. The probability to find an electron with $m' = -1$ at site A and the other electron with $m' = 0$ at site B is given by $\frac{1}{2}(ae + db)^2$. All possible combinations for detectable combinations follow directly from Eq. 4 and are summarized in Table S1 using the C-coefficients. The C-coefficients model the occupation of the entangled m'-orbitals of the Bell-like state $|\Psi^+_{-00-}\rangle$ and are expressed via the coefficients a, b, c, d, e, and f. The C- coefficients are defined by $C_{xy} := \left|\langle m'_y|_B \langle m'_x|_A |\Psi^+_{-00-}\rangle\right|^2$. Here, x and y can be -1, 0 or 1. The values for the C-coefficients as a function of γ are shown in Fig. S2B.

The occupation of $|m'_{-1}\rangle$ at site A without any restriction on the wave function at site B is given by $C_{--} + C_{-0} + C_{-+}$. In full analogy the occupation of $|m'_{-1}\rangle$ at site B is $C_{--} + C_{0-} + C_{+-}$. Due to symmetry we know that $C_{--} + C_{-0} + C_{-+} = C_{--} + C_{0-} + C_{+-}$. Accordingly, the occupation of $|m'_0\rangle$ at site A is given by $C_{0-} + C_{00} + C_{0+}$ and that of $|m'_{+1}\rangle$ is $C_{+-} + C_{+0} + C_{++}$. The dependence of the occupation of the m'-states at site A as a function of γ is shown in Fig. S2C. It should be noted that $C_{--} + C_{-0} + C_{-+} = \frac{1}{2}(ad + da)^2 + \frac{1}{2}(ae + db)^2 + \frac{1}{2}(af + dc)^2 = \frac{1}{2}(a^2 + d^2)$ since $a^2 + b^2 + c^2 = d^2 + e^2 + f^2 = 1$ due to normalization and $a^2d^2 + aedb + afdc = 0$ since $ad + be + cf = 0$ because $|m_{-1}\rangle$ and $|m_0\rangle$ are orthogonal (see Eq. 2 and Eq. 3). The relation $C_{--} + C_{-0} + C_{-+} = \frac{1}{2}(a^2 + d^2)$ shows that for single ionization the entangled state does not produce deviating predictions compared to the classically correlated state for small values of p_- (see below).

The probability to ionize the Bell-like state $|\Psi^+_{-00-}\rangle$ at site A and not ionize it at site B is given by Eq. 5 using the definitions $\overline{p_-} = 1 - p_-$, $\overline{p_0} = 1 - p_0$ and $\overline{p_+} = 1 - p_+$.

$$P^{single\_A}_{-00-} = p_-(\overline{p_-}C_{--} + \overline{p_0}C_{-0} + \overline{p_+}C_{-+}) + p_0(\overline{p_-}C_{0-} + \overline{p_0}C_{00} + \overline{p_+}C_{0+}) + p_+(\overline{p_-}C_{+-} + \overline{p_0}C_{+0} + \overline{p_+}C_{++}) \quad (5)$$

For the $|\Psi^+_{+00+}\rangle$ state we can use the symmetry properties and express the ionization probability with the same C-coefficients. We just have to interchange p_- and p_+. Thus, in full analogy, the single ionization of the Bell-like state $|\Psi^+_{+00+}\rangle$ at site A can be expressed by:

$$P^{single\_A}_{+00+} = p_+(\overline{p_+}C_{--} + \overline{p_0}C_{-0} + \overline{p_-}C_{-+}) + p_0(\overline{p_+}C_{0-} + \overline{p_0}C_{00} + \overline{p_-}C_{0+}) + p_-(\overline{p_+}C_{+-} + \overline{p_0}C_{+0} + \overline{p_-}C_{++}) \quad (6)$$

The probability to singly ionize the Bell-like state at site A or B, but not ionize it at site A and B, is simply two times the probability to ionize it at site A only and given by $P_{-00-}^{single} = 2 \cdot P_{-00-}^{single\_A}$ and $P_{+00+}^{single} = 2 \cdot P_{+00+}^{single\_A}$.

For the description of P_{-00-}^{single} and P_{+00+}^{single} the pump step (with a given helicity) perfectly populates one of the two possible Bell-like states as illustrated in Fig. 1. There are just two possible dissociating states $|\Psi_{-00-}^{+}\rangle$ and $|\Psi_{+00+}^{+}\rangle$ that are relevant in our experiment because we only analyze events for which the molecules dissociate via the $1^3\Pi_u$-state into two ground state oxygen atoms with a KER of about 4 eV (see description of the preparation of the Bell-like state by the pump pulse and Ref. (25)).

Due to symmetry, we know that for $\gamma = 90°$ there must be an equal probability to populate $|\Psi_{-00-}^{+}\rangle$ and $|\Psi_{+00+}^{+}\rangle$. Further, it is expected that the strongest preference to prepare $|\Psi_{-00-}^{+}\rangle$ exists for $\gamma = 0°$. To model this, we assume that the pump pulse populates a virtual state with $m = -1$ or $m = +1$ and that this virtual state is projected to the $1^3\Pi_u$-state.

The sign of m depends on the helicity of the pump pulse. The virtually excited state has a quantization axis that is given by the light's propagation direction since it is populated by absorbing three photons from the pump pulse. In contrast, the quantization axis of the dissociating state $1^3\Pi_u$ is defined by the former molecular axis. The coefficient a [c] models the projection of an $m = -1$ [$m = +1$] orbital onto the quantization axis of the molecular state in full analogy to the previous discussion of the coefficients a, b, c, d, e, and f.

Accordingly, the probability to prepare the state $|\Psi_{-00-}^{+}\rangle$ is modeled by $q_{eff}(\gamma) = \frac{a^2}{a^2+c^2}$ and the probability to prepare the state $|\Psi_{+00+}^{+}\rangle$ is modeled by $1 - q_{eff}(\gamma) = \frac{c^2}{a^2+c^2}$. Using this approach, the expected boundary conditions are fulfilled: $q_{eff}(90°) = 0.5$, $q_{eff}(\gamma - 90°)$=$q_{eff}(90° - \gamma)$, and $q_{eff}(0°) = q_{eff}(180°) = 1$. Further, q_{eff} is monotonic between 0° and 90°. Moreover, we use the parameter $\eta \in [0,0.5]$ to characterize the m-selectivity of the pump step. $\eta = 0$ would indicate that molecules that are aligned along the light propagation axis are always dissociating as illustrated in Fig. 1. $\eta = 0.5$ would indicate that the pump step produces $|\Psi_{-00-}^{+}\rangle$ and $|\Psi_{+00+}^{+}\rangle$ with equal probabilities for all γ (also see Ref. (24)). By using $\tilde{q}_{eff}(\gamma) = 2 \cdot q_{eff}(\gamma) \cdot (0.5 - \eta) + \eta$ instead of $q_{eff}(\gamma)$ we model the γ-dependent pump- selectivity (e.g. for $\eta = 0.263$ the values for $\tilde{q}_{eff}(\gamma)$ are in the range between $\tilde{q}_{eff}(90°) = 0.5$ and $\tilde{q}_{eff}(0°) = 0.737$).

So far, p_- and η are the only free parameters of our model. In the following we will introduce two more free parameters. In analogy to Ref. (41) we use β and additionally κ (to take multiphoton absorption into account) in order to model the γ-dependent dissociation probability:

$$D(\gamma) = \left(1 - 0.25 \cdot \beta \cdot (3 \cdot (sin(\gamma))^2 - 1)\right) \cdot \left(sin(\gamma)\right)^{\kappa} \quad (7)$$

Within our quantum-mechanical model, that uses entangled states, the probability to ionize only one of the two oxygen atoms is:

$$P_{pump\ minus}^{single}(\gamma) = D(\gamma) \cdot \left(\tilde{q}_{eff}(\gamma) \cdot P_{-00-}^{single}(\gamma) + \left(1 - \tilde{q}_{eff}(\gamma)\right) \cdot P_{+00+}^{single}(\gamma)\right) \quad (8)$$

$$P_{pump\ plus}^{single}(\gamma) = D(\gamma) \cdot \left(\tilde{q}_{eff}(\gamma) \cdot P_{+00+}^{single}(\gamma) + \left(1 - \tilde{q}_{eff}(\gamma)\right) \cdot P_{-00-}^{single}(\gamma)\right) \quad (9)$$

All four free parameters are obtained by comparing the result from Eq. 8 and 9 to the experimental data for the ionization of only one of the two oxygen atoms in Fig. 3A. We optimize p_- and η in an outer loop such that the scalar value $\frac{\int P_{pump\ minus}^{single}(\gamma) \cdot sin(\gamma) d\gamma}{\int P_{pump\ plus}^{single}(\gamma) \cdot sin(\gamma) d\gamma}$ matches the experimental finding of 1.07 and that $R_{hightheo} = 0.5$ (see Ref. (24) for details on $R_{hightheo}$). In an inner loop we adjust β and κ making sure that the mean of $P_{pump\ minus}^{single}(\gamma)$ and $P_{pump\ plus}^{single}(\gamma)$ agrees with the mean of the two experimental curves in Fig. 3A (regardless of an overall normalization factor). The value for $R_{hightheo}$ is calculated via $R_{hightheo} = \frac{\int_{\gamma=0°}^{48°} P_{pump\ minus}^{single}(\gamma) \cdot sin(\gamma) d\gamma}{\int_{\gamma=0°}^{48°} P_{pump\ plus}^{single}(\gamma) \cdot sin(\gamma) d\gamma}$ by setting $p_- = 0$, $p_0 = 0$ and $p_+ = 1$. The usage of $R_{hightheo}$ is in full analogy to the procedure used in Ref. (24) and builds on the assumption that for very high electron energies only electrons with $m = +1$ contribute to the electron energy spectrum. The value of $R_{high} = 0.5$ is obtained from the experimental data shown in Fig. S3C, S3F. We obtain the values $p_- = 0.32$, $\eta = 0.263$, $\beta = 1.09$ and $\kappa = 1.87$. The result for $P_{pump\ minus}^{single}$ [$P_{pump\ plus}^{single}$] is plotted as solid blue [red] line in Fig. 3B.

<u>Quantitative modeling of the ionization of both oxygen atoms using entangled states</u>

In full analogy to the derivation of P_{-00-}^{single} and P_{+00+}^{single}, one can express the probability for the ionization of both atoms. The probability to singly ionize the atom at site A and at site B for the Bell-like state $|\Psi_{-00-}^{+}\rangle$ is described by:

$$P_{-00-}^{double} = p_-(p_-C_{--} + p_0C_{-0} + p_+C_{-+}) + p_0(p_-C_{0-} + p_0C_{00} + p_+C_{0+}) + p_+(p_-C_{+-} + p_0C_{+0} + p_+C_{++}) \quad (10)$$

Accordingly, the probability to singly ionize both atoms of the Bell-like state $|\Psi_{+00+}^{+}\rangle$ is given by:

$$P_{+00+}^{double} = p_+(p_+C_{--} + p_0C_{-0} + p_-C_{-+}) + p_0(p_+C_{0-} + p_0C_{00} + p_-C_{0+}) + p_-(p_+C_{+-} + p_0C_{+0} + p_-C_{++}) \quad (11)$$

Within our model, that uses entangled states, the probability to ionize both of the two oxygen atoms is:

$$P^{double}_{pump\ minus}=D(\gamma)\cdot\left(\tilde{q}_{eff}(\gamma)\cdot P^{double}_{-00-}(\gamma)+\left(1-\tilde{q}_{eff}(\gamma)\right)\cdot P^{double}_{+00+}(\gamma)\right) \tag{12}$$

$$P^{double}_{pump\ plus}=D(\gamma)\cdot\left(\tilde{q}_{eff}(\gamma)\cdot P^{double}_{+00+}(\gamma)+\left(1-\tilde{q}_{eff}(\gamma)\right)\cdot P^{double}_{-00-}(\gamma)\right) \tag{13}$$

The result from Eq. 12 [Eq. 13] is shown as solid blue [red] line in Fig. 3D. The model, that uses entangled states, shows very good agreement with the experimental data presented in Fig. 3C. It is important to note that our model has four free parameters ($p_- = 0.32, \eta = 0.263, \beta = 1.09$ and $\kappa = 1.87$). All four free parameters are determined using the experimental results for the single ionization of the Bell-like state. Further, we note that the contributions from $|\Psi^+_{-00-}\rangle$ and $|\Psi^+_{+00+}\rangle$ to the total ionization probability are added up incoherently within our model (in full analogy to Ref. (*24*)). The states $|\Psi^+_{-00-}\rangle$ and $|\Psi^+_{+00+}\rangle$ experience different phase shifts during the preparation by the pump pulse such that the relative phase is random. These phase shifts are due to the AC stark shift (*42*, *43*) that is sensitive to the sign of the magnetic quantum number. The dynamic energy shift is on the order of 1 eV (*44*) and is present during the preparation by the pump pulse for tens of femtoseconds. Accordingly, the contribution from $|\Psi^+_{-00-}\rangle$ and $|\Psi^+_{+00+}\rangle$ can be modeled to be incoherent (also see discussion of $\tilde{q}_{eff}(\gamma)$)."

Quantitative modeling using classically correlated states

For comparison with the model that uses entangled states we use another model, which we refer to as classical model. The classical model uses a classical state which is in 50% of the cases described by $|m_{-1}\rangle_A|m_0\rangle_B$ and in the other 50% it is described by $|m_0\rangle_A|m_{-1}\rangle_B$. (The classical model for $|\Psi^+_{+00+}\rangle$ is defined accordingly.) Besides this difference regarding the definition of the initial state, the classical model is the same as the model that uses entangled states. In full analogy, this leads to nine different C-coefficients $C^{classical}_{xy} := \frac{1}{2}\left|\langle m'_y|_B\langle m'_x|_A|m_{-1}\rangle_A|m_0\rangle_B\right|^2 + \frac{1}{2}\left|\langle m'_y|_B\langle m'_x|_A|m_0\rangle_A|m_{-1}\rangle_B\right|^2$. These classical C-coefficients are used in full analogy to the previously defined C-coefficients and lead to the results that are shown as dashed lines in Fig. 3B, 3D. The usage of a classical state instead of $|\Psi^+_{-00-}\rangle$ or $|\Psi^+_{+00+}\rangle$ excludes entanglement and limits the correlations to classical correlations. The sum over all nine $C^{\text{classical}}$-coefficients is 1 for all values of γ, which ensures that also in the classical model the normalization does not depend on γ. The parameters for the classical model are determined in the same way as for the model that uses entangled states and found to be $p_- = 0.6, \eta = 0.283, \beta = 1.09$ and $\kappa = 1.99$. (As for the model that includes entanglement, also here, the four parameters are determined using only the data from the events that are shown in Fig. 3A.)

The predictions of the classical model for the single ionization of only one of the two atoms is shown by dashed lines in Fig. 3B and shows only minute differences compared to the quantum-mechanical prediction. The differences of the model with entanglement and the classical model for the single ionization of only one of the two atoms would vanish if p_- approached zero.
The classical model for the single ionization of both atoms is realized as described above by using the $C^{\text{classical}}$-coefficients instead of the C-coefficients. The corresponding results from the classical

model are shown as dashed lines in Fig. 3D and show inferior agreement with the experimental results (Fig. 3C) in comparison with the model that includes entanglement.

Spin-orbit dynamics of entangled atoms

For the dissociative $1^3\Pi_u$-state, the good quantum numbers are Ω and Λ. Modeling this state as a linear combination of atomic orbitals leads to $m_{lA} + \mathrm{m_{lB}} = \Lambda$. Here, m_{lA} and m_{lB} are the magnetic quantum numbers of the fourth electrons (see orange arrows in Fig. 2B). Further, we also know that $m_{jA} + m_{jB} = \Omega$. Here, m_{jA} and m_{jB} are the projections of the orbital angular momenta of the two relevant electrons.
It is important to realize that non-adiabatic tunneling is m_l-selective and we can only prepare $\Lambda = -1$ or $\Lambda = +1$. At the time of preparation t_0 we obtain for $\Lambda = -1$ the combination $m_{lA} = 0$ and $m_{lB} = -1$ or the combination of $m_{lA} = -1$ and $m_{lB} = 0$.
Ω can have three different values which are $\Omega = -2$, $\Omega = -1$, $\Omega = 0$ (*45*). For each Ω there are two cases. For $\Omega = -2$ and $\Lambda = -1$ the two cases are as follows. In case 1 we have $m_{jA} = -1$ and $m_{jB} = -1$. In case 2 we have $m_{jA} = 0$ and $m_{jB} = -2$ (or $m_{jA} = -2$ and $m_{jB} = 0$). Here, we exemplarily investigate case 1. At the time of preparation, we prepare $m_{lA} + m_{sA} = m_{jA} = -1$ and $m_{lB} + m_{sB} = m_{jB} = -1$. Knowing that $m_{lA} + \mathrm{m_{lB}} = \Lambda = -1$ results in an entangled state at the time of preparation that is given by $|\Psi^{+}_{-00-}\rangle = \frac{1}{\sqrt{2}}\left(|m_j = -1, m_l = -1, m_s = 0\rangle_A |m_j = -1, m_l = 0, m_s = -1\rangle_B + s|m_j = -1, m_l = 0, m_s = -1\rangle_A |m_j = -1, m_l = -1, m_s = 0\rangle_B\right)$.
Here, $s = 1$ because the symmetry of the wave function in position space is determined by the symmetry of the spin wave function. It can be seen from the Clebsch-Gordan coefficients that the spin wave function is antisymmetric whenever two atomic triplets (e.g. ^{3}P) form a molecular triplet (e.g. $1^3\Pi_u$). This is also the reason why the relative phase ϕ of the Bell-like state is $\phi = 0$ after preparation by the pump pulse (see discussion of Fig. S4 and note that $\phi = 0$ corresponds to $s = e^{i\phi} = 1$).

In a next step, we use that $|m_j = -1, m_l = -1, m_s = 0\rangle$ can be expressed using the available eigenstates (*37*):

$$|m_j = -1, m_l = -1, m_s = 0\rangle = \frac{1}{\sqrt{2}}(|^3P_1\rangle - |^3P_2\rangle) \quad (14)$$

We now use a vector with three entries to indicate the density of the wave function in the three eigenstates $^3P_0$, $^3P_1$, $^3P_2$:

$$|m_j = -1, m_l = -1, m_s = 0\rangle = \frac{1}{\sqrt{2}}\begin{pmatrix} 0 \\ 1 \\ -1 \end{pmatrix} \quad (15)$$

In full analogy we obtain:

$$|m_j = -1, m_l = 0, m_s = -1\rangle = \frac{1}{\sqrt{2}}\begin{pmatrix} 0 \\ -1 \\ -1 \end{pmatrix} \quad (16)$$

This allows to rewrite $|\Psi^{+}_{-00-}\rangle$ as:

$$|\Psi^{+}_{-00-}\rangle = \frac{1}{\sqrt{8}}\left(\begin{pmatrix} 0 \\ 1 \\ -1 \end{pmatrix}_{\mathrm{A}} \begin{pmatrix} 0 \\ -1 \\ -1 \end{pmatrix}_{\mathrm{B}} + \begin{pmatrix} 0 \\ -1 \\ -1 \end{pmatrix}_{\mathrm{A}} \begin{pmatrix} 0 \\ 1 \\ -1 \end{pmatrix}_{\mathrm{B}}\right) \quad (17)$$

The time dependent Bell-like state is then given by:

$$|\Psi(\mathrm{t})\rangle = \begin{pmatrix} e^{-\Delta E_{20} t/\hbar} \\ e^{-\Delta E_{21} t/\hbar} \\ e^{-\Delta E_{22} t/\hbar} \end{pmatrix} |\Psi^{+}_{-00-}\rangle \qquad (18)$$

Here, ΔE_{ij} is the energy difference between ${}^3P_i$ and ${}^3P_j$. The occupation of the originally prepared eigenstate is given by $Q^{\Omega=-2,\mathrm{case1}}_{-00-} = |\langle\Psi^{+}_{-00-}|\Psi(\mathrm{t})\rangle|^2$. The result for $Q^{\Omega=-2,\mathrm{case1}}_{-00-}$ is shown in Fig. S5A.

For each Ω there are two cases. The incoherent sum of all these six cases yields Fig. S5B. The Gaussian peak in Fig. S5B models the distribution of pump-probe time delays in our experiment. The width is 20 fs since we assume that the non-linear dynamics that are driven by pump and probe occur within about 10 fs for each pulse, because the preparation and detection are both highly nonlinear in the intensity of the laser pulses (which have durations of about 40 fs). Weighting the occupations in Fig. S5B with the Gaussian distribution shows that in our measurement 95% of the dissociating atoms with a KER of about 4 eV are in the $|\Psi^{+}_{-00-}\rangle$ state for $\Lambda = -1$. In full analogy this allows one to conclude that 95% of the dissociating atoms are in the $|\Psi^{+}_{+00+}\rangle$ state for $\Lambda = +1$.

Please note, that in Fig. S5A the time after dissociation ΔT is indicated. In our experiment we vary the pump-probe delay T. The time delay that belongs to maximal contrast between the two helicity settings was T=1533 fs. The model shown in Fig. S5B predicts a maximum contrast at ΔT=1468 fs. The difference of T and ΔT is 65 fs. This additional time of 65 fs arises since the molecule takes time to dissociate into atomic states.

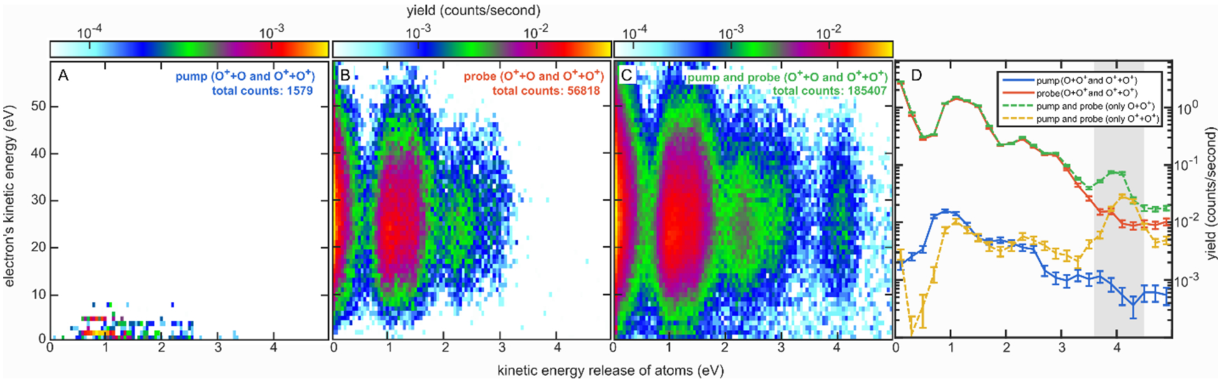

Fig. S1. Experimental details regarding the pump-probe scheme. (A) The experimentally measured rate to detect at least one oxygen ion is shown as a function of the kinetic energy of the detected electron and the kinetic energy release (KER) of the atoms for the case in which only the pump pulse is applied (acquisition time: 274 minutes). **(B)** The same as (A) but here only the probe pulse is applied (acquisition time: 88 minutes). **(C)** The same as (A, B) but here the pump and the probe pulse are both applied (acquisition time: 260 minutes). At a KER of about 4 eV a new feature appears, which is not visible in neither (A) nor (B) and therefore must result from a neutral, dissociating state which was prepared by the pump pulse and was subsequently ionized by the probe pulse. **(D)** The blue and red line show the same data as in (A, B**)**. The yellow line shows the experimentally determined rate for the ionization of both oxygen atoms by the probe pulse. The green line shows the experimentally determined rate for the ionization of one of the two oxygen atoms by the probe pulse after subtracting random coincidences, that are due to cases in which both oxygen atoms are ionized but only one is detected (see text for details). All data shown here were measured using a pump pulse with an anticlockwise rotating laser electric field (corresponds to predominantly preparing $|\Psi^{+}_{-00-}\rangle$). The gray shaded area in (D) highlights the energy range around a KER of 4 eV. It should be noted that the events with a KER that is smaller than 3.5 eV mainly belong to the production of O^+ + O and the events with a KER that is higher than 3.5 eV belong to the production of $O^+ + O^+$. The channel $O^+ + O^+$ in (B, C) has another pronounced maximum at a KER of 7.6 eV (not shown, see text for details). Also see Fig. S3 for details regarding the electron's energy spectra.

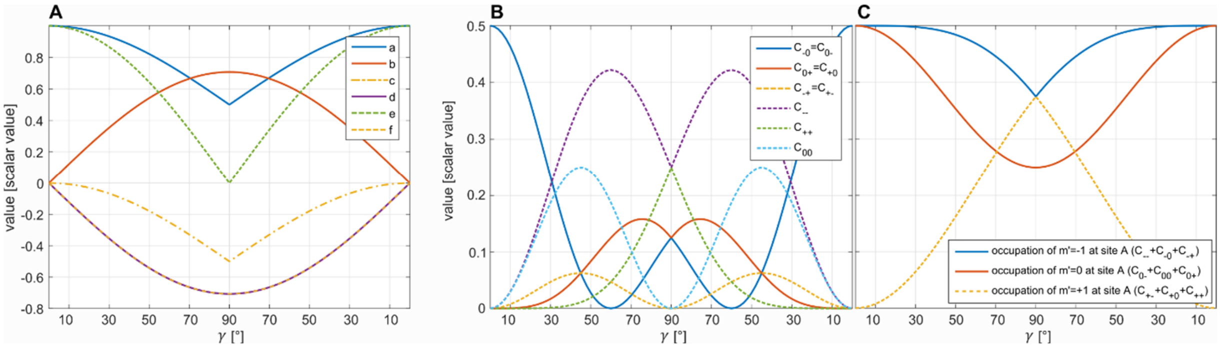

Fig. S2. Calculated coefficients that are used to project the prepared state to a new quantization axis. (A) Values of the coefficients a, b, c, d, e, and f are shown as a function of γ, which is the angle between the former molecular axis and the light's propagation axis. The coefficients are used to express the Bell-like state using the quantization axis of the probe pulse. **(B)** Values of the C-coefficients that represent the occupation of the different entangled m'-orbitals. The sum over the nine C-coefficients is 1 for all values of γ. **(C)** Occupation of the different uncorrelated m'-orbitals.

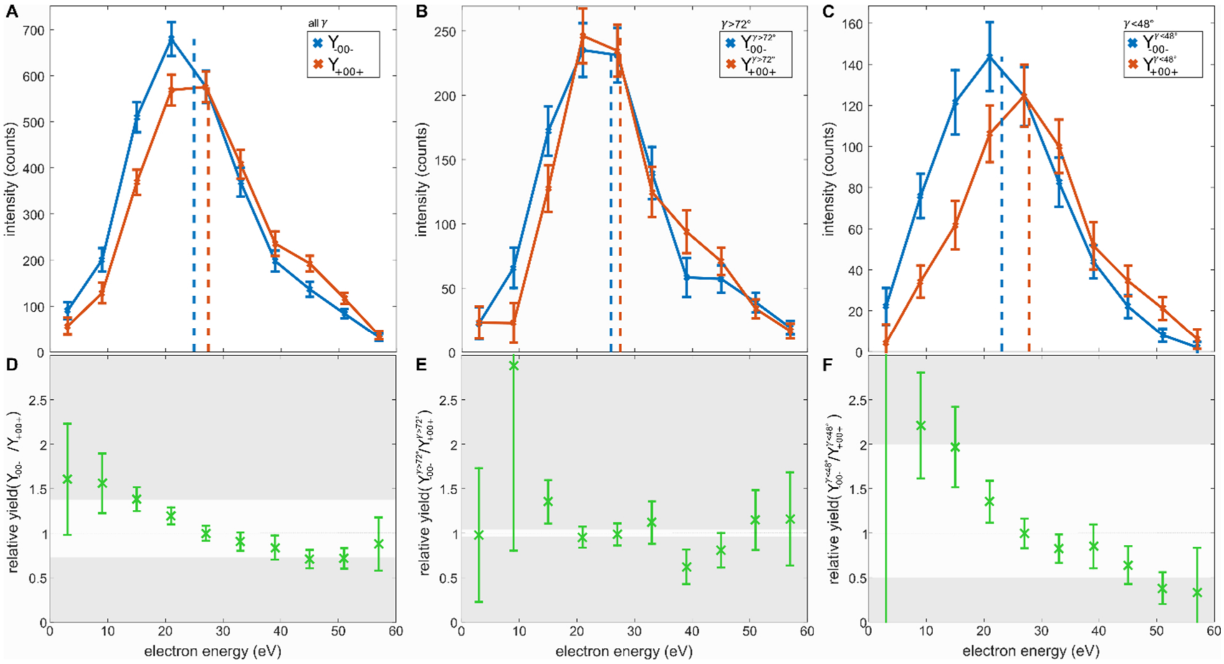

Fig. S3. Experimental details on the pump efficiency. (A) Y_{-00-} [Y_{+00+}] is the experimentally obtained electron energy spectrum that is measured in coincidence with the data that is depicted in blue [red] in Fig. 3A. **(B)** [**(C)**] The same as (A) but for the subset of events with $\gamma > 72°$ [$\gamma < 48°$]. The vertical dashed lines in (A-C) indicate the corresponding mean values of the measured electron energy distributions. **(D-F)** The ratios Y_{-00-}/Y_{+00+} from the data that is shown in (A-C). It is seen in (F) that the ratio reaches values down to $R_{high} = 0.5$ for high electron energies (see text). The gray shaded areas in (D-F) indicate ratios that are not expected for an entangled state within our quantum mechanical model for the parameters that are used for the solid line in Fig. 3B and 3D ($p_- = 0.32, \eta = 0.263, \beta = 1.09$ and $\kappa = 1.87$).

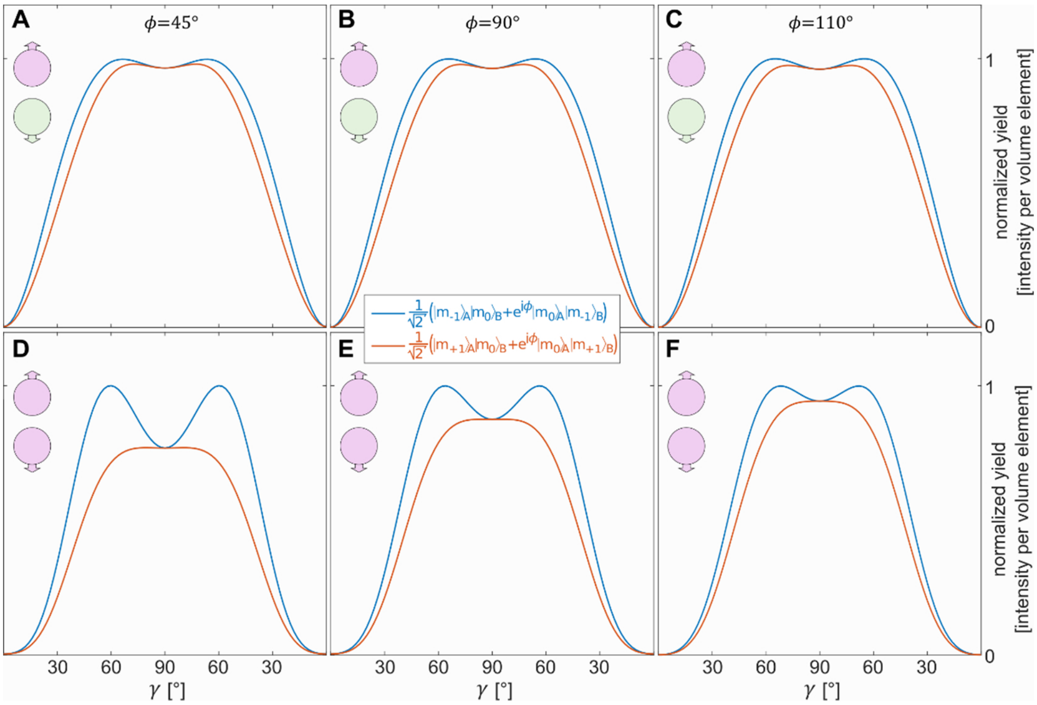

Fig. S4. Details on the phase ϕ of the Bell-like state. **(A)** The results from the quantum-mechanical model (in analogy to the solid lines in Fig. 3B) but here the initial state $\frac{1}{\sqrt{2}}(|m_{-1}\rangle_A|m_0\rangle_B + e^{i\phi}|m_0\rangle_A|m_{-1}\rangle_B)$ is used for the blue line and the initial state $\frac{1}{\sqrt{2}}(|m_{+1}\rangle_A|m_0\rangle_B + e^{i\phi}|m_0\rangle_A|m_{+1}\rangle_B)$ is used for the red line with a phase of $\phi = 45°$. For this scenario the parameters $p_{-} = 0.37, \eta = 0.266, \beta = 1.09$ and $\kappa = 1.89$ are found using the same procedure as for Fig. 3. **(B)** The same as (A) but a phase of $\phi = 90°$ is used and the parameters $p_{-} = 0.6, \eta = 0.283, \beta = 1.09$ and $\kappa = 1.99$ are found. This result is equivalent to the result for classically correlated states (compare dashed lines in Fig. 3B). **(C)** The same as (A) but a phase of $\phi = 110°$ is used and the parameters $p_{-} = 0.84, \eta = 0.302, \beta = 1.08$ and $\kappa = 2.11$ are found. (A-C) show results for the single ionization of one of the two atoms (in analogy to the solid lines in Fig. 3B) and (D-F) show results for the single ionization of both of the two atoms of the Bell-like state (in analogy to the solid lines in Fig. 3D). For (D) the same parameters (ϕ, p_{-}, η, β and κ) are used as in (A). In (E) [(F]) the same parameters are used as in (B) [(C)].

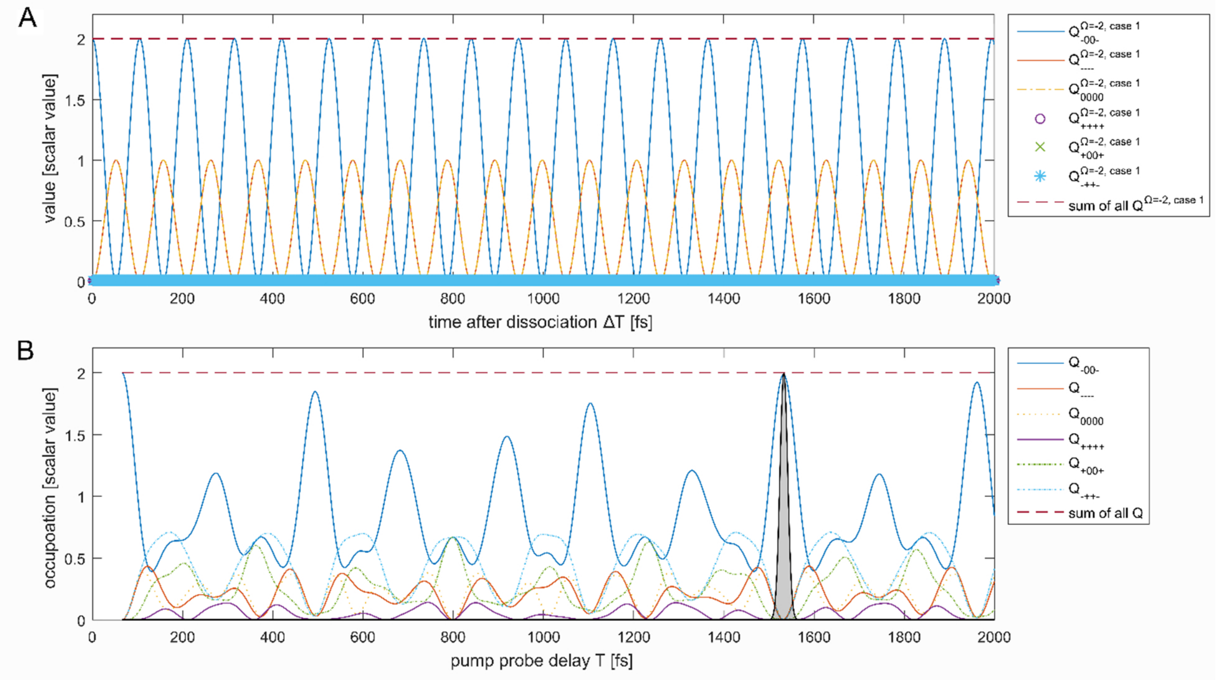

Fig. S5. **Spin-orbit dynamics after dissociation of the oxygen molecules by the pump pulse. (A)** $Q_{-00-}^{\Omega=-2,\mathrm{case1}}$ shows the occupation of the originally prepared eigenstate (see text) for $m_{jA} = -1$, $m_{jB} = -1$ and $\Omega = -2$. **(B)** Q_{-00-} shows the occupation of the originally prepared eigenstate as a function of the pump-probe delay T. The gray shaded region indicates a Gaussian distribution with a full-width-at-half-maximum of 20 fs that is centered at 1533 fs. In addition, the occupations of the other entangled states are shown in full analogy. Please note that in (A) the time after dissociation ΔT is indicated. In our experiment we vary the pump-probe delay $T = \Delta T$+65 fs that is shown in (B) (see text).

	$\lvert m'_{-1}\rangle_A$	$\lvert m'_{0}\rangle_A$	$\lvert m'_{+1}\rangle_A$
$\lvert m'_{-1}\rangle_B$	$C_{--} = \frac{1}{2}\lvert ad + da\rvert^2$	$C_{0-} = \frac{1}{2}\lvert bd + ea\rvert^2$	$C_{+-} = \frac{1}{2}\lvert cd + fa\rvert^2$
$\lvert m'_{0}\rangle_B$	$C_{-0} = \frac{1}{2}\lvert ae + db\rvert^2$	$C_{00} = \frac{1}{2}\lvert be + eb\rvert^2$	$C_{+0} = \frac{1}{2}\lvert ce + fb\rvert^2$
$\lvert m'_{+1}\rangle_B$	$C_{-+} = \frac{1}{2}\lvert af + dc\rvert^2$	$C_{0+} = \frac{1}{2}\lvert bf + ec\rvert^2$	$C_{++} = \frac{1}{2}\lvert cf + fc\rvert^2$

Table S1. Overview of the C-coefficients. The C-coefficients are based on Eq. 4. They express the occupation of the different entangled m'-orbitals (see Eq. 5, 6, 10 and 11) using the coefficients a, b, c, d, e, and f (see Fig. S2).

REFERENCES

1. S. L. Chin, F. Yergeau, P. Lavigne, Tunnel ionisation of Xe in an ultra-intense CO_2 laser field (10^{14} W cm^{-2}) with multiple charge creation. *J. Phys. B.* **18**, L213–L215 (1985).

2. P. B. Corkum, Plasma perspective on strong field multiphoton ionization. *Phys. Rev. Lett.* **71**, 1994–1997 (1993).

3. L. V. Keldysh, Ionization in the field of a strong electromagnetic wave. *Sov. Phys. JETP.* **20**, 1307–1314 (1965).

4. P. Eckle, A. N. Pfeiffer, C. Cirelli, A. Staudte, R. Dörner, H. G. Muller, M. Büttiker, U. Keller, Attosecond ionization and tunneling delay time measurements in helium. *Science* **322**, 1525–1529 (2008).

5. E. Hagley, X. Maître, G. Nogues, C. Wunderlich, M. Brune, J. M. Raimond, S. Haroche, Generation of Einstein-Podolsky-Rosen pairs of atoms. *Phys. Rev. Lett.* **79**, 1 (1997), 5.

6. J. S. Bell, On the Einstein Podolsky Rosen Paradox. *Phys. Phys. Fiz.* **1**, 195–200 (1964).

7. E. Schrödinger, Die gegenwärtige Situation in der Quantenmechanik. *Naturwissenschaften* **23**, 807–812 (1935).

8. M. J. J. Vrakking, Control of attosecond entanglement and coherence. *Phys. Rev. Lett.* **126**, 113203 (2021).

9. A. Einstein, B. Podolsky, N. Rosen, Can quantum-mechanical description of physical reality be considered complete? *Phys. Rev.* **47**, 777–780 (1935).

10. M. Born, Metaphysical conclusions, in *Natural Philosophy of Cause and Chanc*e (Clarendon Press, Oxford, 1949), pp. 122.

11. J. F. Clauser, A. Shimony, Bell's theorem. Experimental tests and implications. *Rep. Prog. Phys.* **41**, 1881–1927 (1978).

12. A. Aspect, P. Grangier, G. Roger, Experimental tests of realistic local theories via Bell's theorem. *Phys. Rev. Lett.* **47**, 460–463 (1981).

13. A. Aspect, P. Grangier, G. Roger, Experimental realization of Einstein-Podolsky-Rosen-Bohm Gedankenexperiment: A new violation of Bell's inequalities. *Phys. Rev. Lett.* **49**, 91–94 (1982).

14. J. I. Cirac, P. Zoller, Quantum computations with cold trapped ions. *Phys. Rev. Lett.* **74**, 4091–4094 (1995).

15. S. J. Freedman, F. Clauser, Experimental test of local hidden-variable theories. *Phys. Rev. Lett.* **28**, 938–941 (1972).

16. J. W. Pan, D. Bouwmeester, H. Weinfurter, A. Zeilinger, Experimental entanglement swapping: Entangling photons that never interacted. *Phys. Rev. Lett.* **80**, 3891–3894 (1998).

17. Q. A. Turchette, C. S. Wood, B. E. King, C. J. Myatt, D. Leibfried, W. M. Itano, C. Monroe, D. J. Wineland, Deterministic entanglement of two trapped ions. *Phys. Rev. Lett.* **81**, 3631–3634 (1998).

18. M. Ivanov, M. Spanner, O. Smirnova, Anatomy of strong field ionization. *J. Mod. Opt.* **52**, 165–184 (2005).

19. U. S. Sainadh, H. Xu, X. Wang, A. Atia-Tul-Noor, W. C. Wallace, N. Douguet, A. Bray, I. Ivanov, K. Bartschat, A. Kheifets, R. T. Sang, I. V. Litvinyuk, Attosecond angular streaking and tunnelling time in atomic hydrogen. *Nature* **568**, 75–77 (2019).

20. J. Ullrich, R. Moshammer, A. Dorn, R. Dörner, L. P. H. Schmidt, H. Schmidt-Böcking, Recoil-ion and electron momentum spectroscopy: Reaction-microscopes. *Rep. Prog. Phys*. **66**, 1463–1545 (2003).

21. R. M. Wood, Q. Zheng, A. K. Edwards, M. A. Mangan, Limitations of the axial recoil approximation in measurements of molecular dissociation. *Rev. Sci. Instrum.* **68**, 1382–1386 (1997).

22. I. Barth, O. Smirnova, Nonadiabatic tunneling in circularly polarized laser fields: Physical picture and calculations. *Phys. Rev. A* **84**, 063415 (2011).

23. T. Herath, L. Yan, S. K. Lee, W. Li, Strong-field ionization rate depends on the sign of the magnetic quantum number. *Phys. Rev. Lett.* **109**, 043004 (2012).

24. S. Eckart, M. Kunitski, M. Richter, A. Hartung, J. Rist, F. Trinter, K. Fehre, N. Schlott, K. Henrichs, L. P. H. Schmidt, T. Jahnke, M. Schöffler, K. Liu, I. Barth, J. Kaushal, F. Morales, M. Ivanov, O. Smirnova, R. Dörner, Ultrafast preparation and detection of ring currents in single atoms. *Nat. Phys.* **14**, 701–704 (2018).

25. R. P. Saxon, B. Liu, Ab initio configuration interaction study of the valence states of O_2. *J. Chem. Phys.* **67**, 5432–5441 (1977).

26. H. M. Lambert, A. A. Dixit, E. W. Davis, P. L. Houston, Quantum yields for product formation in the 120-133 nm photodissociation of O_2. *J. Chem. Phys.* **121**, 10437–10446 (2004).

27. Y. L. Huang, R. J. Gordon, The multiplet state distribution of O(^3P_J) produced in the photodissociation of O_2 at 157 nm. *J. Chem. Phys.* **94**, 2640–2647 (1991).

28. I. Barth, J. Manz, Electric ring currents in atomic orbitals and magnetic fields induced by short intense circularly polarized π laser pulses. *Phys. Rev. A* **75**, 012510 (2007).

29. S. A. Trushin, W. E. Schmid, W. Fu, Time-resolved photodissociation of oxygen at 162 nm. *J. Phys. B.* **44**, 165602 (2011).

30. L. Ding, S. Mardazad, S. Das, S. Szalay, U. Schollwöck, Z. Zimborás, C. Schilling, Concept of orbital entanglement and correlation in quantum chemistry. *J. Chem. Theory Comput.* **17**, 79–95 (2021).

31. J. Li, S. Kais, Entanglement classifier in chemical reactions. *Sci. Adv.* **5**, eaax5283 (2019).

32. M. Ruberti, Onset of ionic coherence and ultrafast charge dynamics in attosecond molecular ionisation. *Phys. Chem. Chem. Phys.* **21**, 17584–17604 (2019).

33. J. Rivera-Dean, T. Lamprou, E. Pisanty, P. Stammer, A. F. Ordóñez, A. S. Maxwell, M. F. Ciappina, M. Lewenstein, P. Tzallas, Strong laser fields and their power to generate controllable high-photon-number coherent-state superpositions. *Phys. Rev. A* **105**, 033714 (2022).

34. M. Lewenstein, M. F. Ciappina, E. Pisanty, J. Rivera-Dean, P. Stammer, T. Lamprou, P. Tzallas, Generation of optical Schrödinger cat states in intense laser–matter interactions. *Nat. Phys.* **17**, 1104–1108 (2021).

35. A. S. Maxwell, L. B. Madsen, M. Lewenstein, Entanglement of orbital angular momentum in non-sequential double ionization. *Nat. Commun.* **13**, 4706 (2022).

36. O. Jagutzki, A. Cerezo, A. Czasch, R. Dörner, M. Hattaß, M. Huang, V. Mergel, U. Spillmann, K. Ullmann-Pfleger, T. Weber, H. Schmidt-Böcking, G. D. W. Smith, Multiple hit

readout of a microchannel plate detector with a three-layer delay-line anode. *IEEE Trans. Nucl. Sci.* **49**, 2477–2483 (2002).

37. A. N. Pfeiffer, S. G. Sayres, S. R. Leone, Calculation of valence electron motion induced by sequential strong-field ionisation. *Mol. Phys.* **111**, 2283–2291 (2013).

38. A. V. Baklanov, L. M. C. Janssen, D. H. Parker, L. Poisson, B. Soep, J. M. Mestdagh, O. Gobert, Direct mapping of recoil in the ion-pair dissociation of molecular oxygen by a femtosecond depletion method. *J. Chem. Phys.* **129**, 214306 (2008).

39. K. Fehre, D. Trojanowskaja, J. Gatzke, M. Kunitski, F. Trinter, S. Zeller, L. P. H. Schmidt, J. Stohner, R. Berger, A. Czasch, O. Jagutzki, T. Jahnke, R. Dörner, M. S. Schöffler, Absolute ion detection efficiencies of microchannel plates and funnel microchannel plates for multi-coincidence detection. *Rev. Sci. Instrum.* **89**, 045112 (2018).

40. I. Barth, O. Smirnova, Nonadiabatic tunneling in circularly polarized laser fields. II. Derivation of formulas. *Phys. Rev. A* **87**, 013433 (2013).

41. J. J. Lin, D. W. Hwang, Y. T. Lee, X. Yang, Photodissociation of O_2 at 157 nm: Experimental observation of anisotropy mixing in the $O_2+h\nu \rightarrow O(^3P)+O(^3P)$ channel. *J. Chem. Phys.* **109**, 1758–1762 (1998).

42. N. B. Delone, V. P. Krainov, AC Stark shift of atomic energy levels. *Phys. Usp.* **42**, 669–687 (1999).

43. A. H. N. C. De Silva, D. Atri-Schuller, S. Dubey, B. P. Acharya, K. L. Romans, K. Foster, O. Russ, K. Compton, C. Rischbieter, N. Douguet, K. Bartschat, D. Fischer, Using circular dichroism to control energy transfer in multiphoton ionization. *Phys. Rev. Lett.* **126**, 023201 (2021).

44. X. Wu, Z. Yang, S. Zhang, X. Ma, J. Liu, D. Ye, Buildup time of Autler-Townes splitting in attosecond transient absorption spectroscopy. *Phys. Rev. A* **103**, L061102 (2021).

45. M. C. G. N. Van Vroonhoven, G. C. Groenenboom, Reassignment of the O_2 spectrum just below dissociation threshold based on ab initio calculations. *J. Chem. Phys.* **117**, 5240–5251 (2002).
